# Specific tRNAs promote mRNA decay by recruiting the CCR4-NOT complex to translating ribosomes

**DOI:** 10.1126/science.adq8587

**Published:** 2024-11-22

**Authors:** Xiaoqiang Zhu, Victor Emmanuel Cruz, He Zhang, Jan P. Erzberger, Joshua T. Mendell

**Affiliations:** 1 Department of Molecular Biology, University of Texas Southwestern Medical Center, Dallas, TX 75390, USA; 2 Department of Biophysics, University of Texas Southwestern Medical Center, Dallas, TX 75390, USA; 3 Quantitative Biomedical Research Center, University of Texas Southwestern Medical Center, Dallas, TX 75390, USA; 4 Department of Clinical Sciences, University of Texas Southwestern Medical Center, Dallas, TX 75390, USA; 5 Harold C. Simmons Comprehensive Cancer Center, University of Texas Southwestern Medical Center, Dallas, TX 75390, USA; 6 Hamon Center for Regenerative Science and Medicine, University of Texas Southwestern Medical Center, Dallas, TX 75390, USA; 7 Howard Hughes Medical Institute, University of Texas Southwestern Medical Center, Dallas, TX 75390, USA

## Abstract

**Introduction:**

The rate of messenger RNA (mRNA) degradation is a major determinant of the amount of protein produced from each gene. Many RNA binding proteins and regulatory RNAs promote mRNA decay by binding to specific messages and recruiting degradation factors, including the CCR4-NOT complex, which destabilizes mRNAs by removing their poly(A) tails. Recent work has revealed that the CCR4-NOT complex can also be directly recruited to ribosomes when translation proceeds inefficiently. Specifically, when a ribosome encounters a codon with limited cognate tRNA, termed a non-optimal codon, it may pause in a conformation with empty A- and E-sites. This enables binding of CNOT3 (known as Not5 in yeast), a subunit of the CCR4-NOT complex, to the vacant E-site, thereby promoting accelerated turnover of the mRNA.

**Rationale:**

Non-optimal codons are the strongest stimuli for ribosomal recruitment of Not5 in yeast, but the sequence features that lead to CNOT3 recruitment to translating ribosomes in mammalian cells have not yet been studied on a transcriptome-wide level. In general, non-optimal codons have a weaker effect on mRNA stability in mammalian cells compared to yeast, suggesting that other determinants may play a more dominant role in CCR4-NOT recruitment in mammals. We therefore applied selective ribosome profiling in mammalian cells to identify the predominant sequence features of mRNAs whose translation leads to ribosomal recruitment of CNOT3.

**Results:**

High-throughput sequencing of mRNA footprints associated with CNOT3-bound ribosomes in HEK293T cells revealed that the presence of slowly-decoded codons in the A-site was not a strong signal for ribosomal recruitment of CNOT3. Instead, specific arginine codons (CGG, CGA, and AGG) in the P-site were highly associated with CNOT3 recruitment, while other codons, including those specifying asparagine, lysine, isoleucine, tyrosine, phenylalanine, methionine, and threonine, were depleted from the P-site of CNOT3-bound ribosomes. Global measurements of mRNA half-lives coupled with analyses of mRNA codon content demonstrated that transcripts encoding mitochondrial ribosomal proteins are highly enriched for CNOT3-associated arginine codons and, accordingly, the CCR4-NOT complex is a strong negative regulator of mitochondrial translation and mass.

To investigate the mechanism by which P-site codon identity regulates CNOT3 recruitment, we performed cryo-electron microscopy (cryo-EM) of CNOT3-bound ribosomes with a CGG arginine codon in the P-site. The resulting high-resolution structures, coupled with tRNA and CNOT3 mutagenesis, uncovered a central role for the P-site tRNA in CNOT3 recruitment. The results demonstrated that CNOT3 enters the empty E-site and engages in hydrogen bonding interactions with the D-arm of tRNA^Arg,CCG^ in the P-site. These interactions, which promote CNOT3 recruitment, are dependent upon the presence of a rare U13:A22:A46 triplet in arginine tRNAs that decode CGG, CGA, and AGG. Furthermore, tRNAs that decode the codons that are depleted from CNOT3-bound ribosomes frequently contain an extra nucleotide in the D-loop that sterically clashes with CNOT3, thereby blocking its recruitment.

**Conclusion:**

Based upon these data, we propose a model for co-translational recruitment of the CCR4-NOT complex in mammals. As previously demonstrated, slow decoding results in a ribosomal conformation with empty A- and E-sites. This enables CNOT3 to enter the vacant E-site and probe the P-site tRNA. Arginine tRNAs containing a U13:A22:A46 triplet engage in stabilizing interactions with CNOT3, thereby promoting mRNA decay, while tRNAs with an extra nucleotide in the D-loop oppose stable CNOT3 binding. These findings reveal that, in addition to their canonical role in decoding, tRNAs participate in the recruitment of post-transcriptional regulators to translating ribosomes. We propose the term P-site tRNA-mediated mRNA decay (PTMD) to describe this mechanism of accelerated mRNA turnover.

## Abstract (summary)

The CCR4-NOT complex is a major regulator of eukaryotic mRNA stability. Slow decoding during translation promotes association of CCR4-NOT with ribosomes, accelerating mRNA degradation. Here, we applied selective ribosome profiling to further investigate the determinants of CCR4-NOT recruitment to ribosomes in mammalian cells. This revealed that specific arginine codons in the P-site are strong signals for ribosomal recruitment of human CNOT3, a CCR4-NOT subunit. Cryo-electron microscopy and tRNA mutagenesis demonstrated that the unique D-arms of select arginine tRNAs interact with CNOT3 and promote its recruitment, while other tRNA D-arms sterically clash with CNOT3. These effects link codon content to mRNA stability. Thus, in addition to their canonical decoding function, tRNAs directly engage regulatory complexes during translation, a mechanism we term P-site tRNA-mediated mRNA decay (PTMD).

## Graphical Abstract

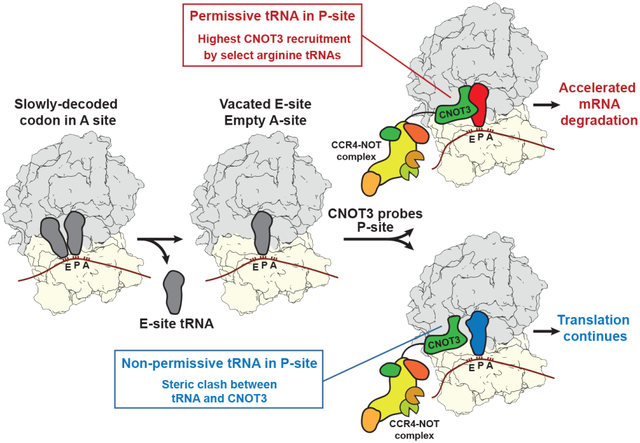

**The P-site tRNA governs recruitment of the CCR4-NOT complex to translating ribosomes.** Slow decoding, resulting in a ribosome with empty A- and E-sites, provides an opportunity for CNOT3 enter the E-site and probe the P-site tRNA. Permissive P-site tRNAs, especially select arginine tRNAs, stabilize CNOT3 recruitment and promote mRNA decay, while other non-permissive tRNAs sterically block CNOT3 association, enabling continued translation.

Precise control of messenger RNA (mRNA) translation and stability plays a central role in establishing appropriate levels of gene expression. These parameters are regulated by the poly(A) tail, which is present on nearly all eukaryotic mRNAs (1). The poly(A) tail is bound by cytoplasmic polyadenylate-binding proteins (PABPCs), which stimulate efficient translation and protect the message from both 5’ and 3’ exonucleolytic degradation (2–4). Opposing these activities is the CCR4-NOT complex, a highly conserved multi-subunit assembly that functions as the major cytoplasmic deadenylase (5–7). mRNA deadenylation rates and half-lives vary by several orders of magnitude (8) and are highly influenced by mechanisms that recruit the CCR4-NOT complex to specific transcripts. For example, many RNA binding proteins (RBPs), such as YTHDF2, Pumilio, and the cytoplasmic polyadenylation element binding (CPEB) proteins, interact with the CCR4-NOT complex, thus accelerating the deadenylation of specific mRNAs to which they are bound (9–12). Likewise, microRNAs (miRNAs) recruit CCR4-NOT to target mRNAs through direct interaction of TNRC6 with CNOT9, core components of the miRNA-induced silencing complex (miRISC) and the CCR4-NOT complex, respectively (13).

The CCR4-NOT complex can also be recruited directly to translating ribosomes to elicit accelerated degradation of specific sets of transcripts (14, 15). In *Saccharomyces cerevisiae*, the rate of decoding is a key determinant of Ccr4-Not engagement with ribosomes (14, 16–18). When a ribosome encounters a non-optimal codon, defined as a codon with a low concentration of cognate tRNA, the E-site tRNA may be released before the A-site codon is decoded. The resulting ribosomal conformation, with empty A- and E-sites, enables the N-terminal helical bundle of Not5, a component of Ccr4-Not and homolog of human CNOT3, to enter the vacant E-site (14). In this manner, the Ccr4-Not complex can monitor the efficiency of decoding, accelerating the degradation of mRNAs enriched in non-optimal codons. Recently, stalling of mammalian ribosomes on a highly non-optimal codon in an *in vitro* rabbit reticulocyte lysate system was shown to similarly lead to recruitment of CNOT3 to empty E-sites (19). Moreover, multiple studies have documented that slow translation elongation, due to codon content or amino acid availability, is associated with accelerated mRNA turnover in mammalian cells (20–26). While these observations suggest a conserved function for CCR4-NOT in monitoring decoding efficiency, the predominant determinants of CNOT3 recruitment to translating ribosomes in mammals have not yet been studied on a transcriptome-wide level.

Here we used selective ribosome profiling in human cells to determine the features of mRNAs whose translation leads to CNOT3 recruitment to ribosomes. We found that the strongest determinant of CNOT3 recruitment was not the decoding efficiency of the codon in the ribosomal A-site, but rather the identity of the codon in the P-site. The presence of select arginine codons in the P-site were particularly strong signals for CNOT3 association, while codons specifying several other amino acids were depleted from the P-site of CNOT3-bound ribosomes. High-resolution cryo-electron microscopy (cryo-EM) structures of CNOT3-bound human ribosomes revealed that these effects were attributable to direct interactions between CNOT3 and the D-arm of the P-site tRNA, which promote or prevent accommodation of CNOT3 in the vacant ribosomal E-site. These findings demonstrate that, in addition to their canonical role in decoding, tRNAs recruit post-transcriptional regulators to translating ribosomes, uncovering a previously unrecognized P-site tRNA-mediated mRNA decay (PTMD) pathway.

## The ribosomal P-site codon is a major determinant of CNOT3 recruitment to human ribosomes

**Fig. 1.**
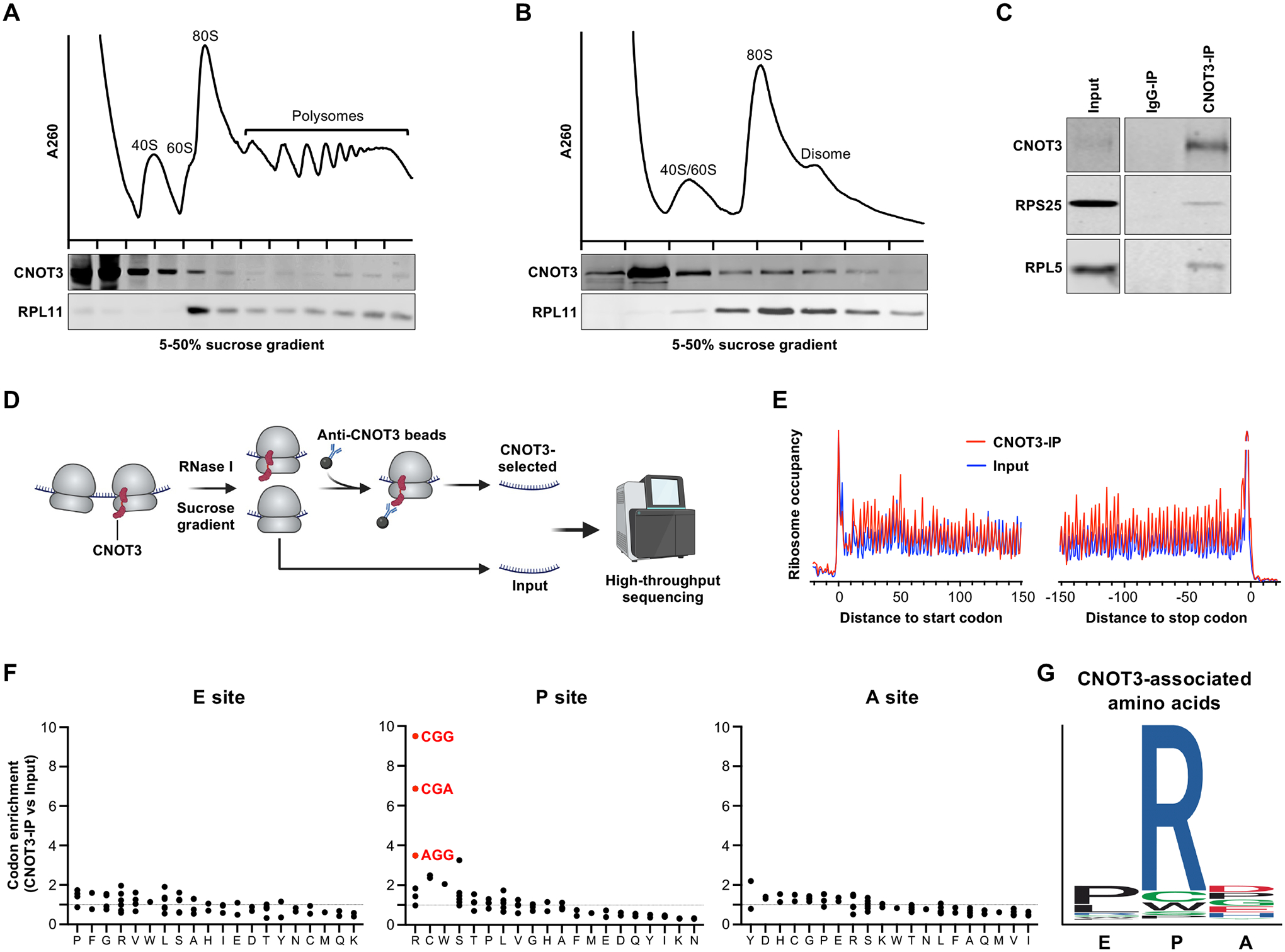
Select arginine codons are enriched in the ribosomal P-site of CNOT3-bound ribosomes. (**A** and **B**) Sucrose density gradient profiles of HEK293T cell lysate and western blot analysis of fractions without (A) or with RNase A treatment (B). (**C**) Western blot analysis of CNOT3 or control immunoprecipitates. Representative results from n=3 biological replicates shown for sucrose density gradient profiles and CNOT3 IP. (**D**) Schematic of CNOT3-selective ribosome profiling. Figure created with BioRender.com. (**E**) Meta-codon plots showing the triplet periodicity of ribosome profiling reads. (**F**) Codon enrichment in ribosomal E, P, and A-sites of CNOT3-bound ribosomes. (**G**) Sequence logo representation of amino acid enrichment in ribosomal E, P, and A-sites of CNOT3-bound ribosomes.

We first examined whether human CNOT3 associates with translating ribosomes using sucrose density gradient ultracentrifugation of HEK293T cell lysates. Similar to the behavior of Not5, the CNOT3 ortholog in *Saccharomyces cerevisiae* (14), endogenous CNOT3, or stably-expressed Flag-tagged CNOT3, co-sedimented with higher order polysomes in human cells (Fig. 1A and fig. S1A). Polysome association of CNOT3 could be due to a direct interaction with translating ribosomes or could occur as a consequence of indirect recruitment to translated mRNAs via RBPs or miRNAs. To distinguish between these possibilities, lysates were treated with RNase prior to sucrose gradient fractionation and CNOT3 was immunoprecipitated from the resulting monosome fraction (Fig. 1, B and C and fig. S1, B and C). CNOT3 co-sedimented with monosomes and stably associated with large and small ribosomal subunit proteins, consistent with direct binding of CNOT3 to ribosomes.

We next performed selective ribosome profiling (27) to identify the features of mRNAs that were associated with CNOT3-bound ribosomes. Monosomes were prepared by RNase treatment and sucrose gradient fractionation, followed by immunoprecipitation (IP) of CNOT3 (Fig. 1D). Sequencing of the enriched ribosomal footprints confirmed the expected triplet periodicity within open reading frames (ORFs) (Fig. 1E and fig. S1, D and E), consistent with the stepwise translation of each codon and enabling assignment of the A-, P-, and E-site codons in each footprint.

In *Saccharomyces cerevisiae*, the Ccr4-Not complex preferentially associates with ribosomes with non-optimal codons in the A site (14). Codon optimality can be quantified using the tRNA adaptation index (tAI), a metric of cognate tRNA abundance (28). Re-analysis of published selective ribosome profiling data from yeast (14) confirmed that non-optimal codons with a low tAI (29) are highly enriched in the A-site of Ccr4-Not-bound ribosomes (fig. S1, F to H). In contrast, we observed no preferential enrichment of non-optimal codons in the A-site of CNOT3-bound human ribosomes and, accordingly, no correlation between tAI (20) and A-site codon enrichment (fig. S1, I to K). Similarly, codons with a low codon stability coefficient (CSC), a metric derived from global mRNA decay rate measurements which reflects whether a given codon has a positive or negative effect on overall mRNA stability (21), were not enriched in the A-site of CNOT3-bound ribosomes (fig. S1, L and M). In addition to tRNA availability, the rate of decoding is also impacted by additional parameters, such as amino acid concentration and tRNA charging (30). The relative decoding rate, which accounts for these additional variables, is approximated by the A-site dwell time of each codon, a parameter that can be estimated using ribosome profiling data (25). We observed a modest, but statistically significant, correlation between A-site dwell time and codon enrichment in the A-site of CNOT3-bound ribosomes (fig. S1N). Nevertheless, no specific A-site codons were strongly enriched in our dataset, with only a single codon exhibiting greater than two-fold enrichment (Fig. 1F). Overall, these data suggest that slow decoding of the A-site codon measurably promotes CNOT3 recruitment to translating ribosomes, but also raise the possibility that other determinants play a more dominant role in co-translational CNOT3 recruitment in mammalian cells.

To more broadly explore a possible connection between codon content and CNOT3 recruitment, we examined codon enrichment in the ribosomal P- and E-sites in our selective ribosome profiling data (Fig. 1F). This revealed that the most enriched codons in CNOT3-bound ribosomes were located in the P-site. In particular, select P-site arginine codons (CGG, CGA, and AGG) were the most enriched codons observed in any position within CNOT3-bound ribosomes, while other arginine codons (CGC, AGA, and CGU) exhibited weak enrichment. Analysis of the amino acids encoded by CNOT3-bound ribosomal footprints further documented a robust enrichment of arginine-centered tripeptides (Fig. 1G), with 18 of the top 20 enriched tripeptides containing arginine at the P-site (fig. S1O). Altogether, these data demonstrate that P-site codon identity is strongly associated with co-translational CNOT3 recruitment in human cells, with select arginine codons at the P-site providing the strongest detectable signal for CNOT3 binding.

## CGG, CGA, and AGG arginine codons promote CNOT3-mediated mRNA decay

**Fig. 2.**
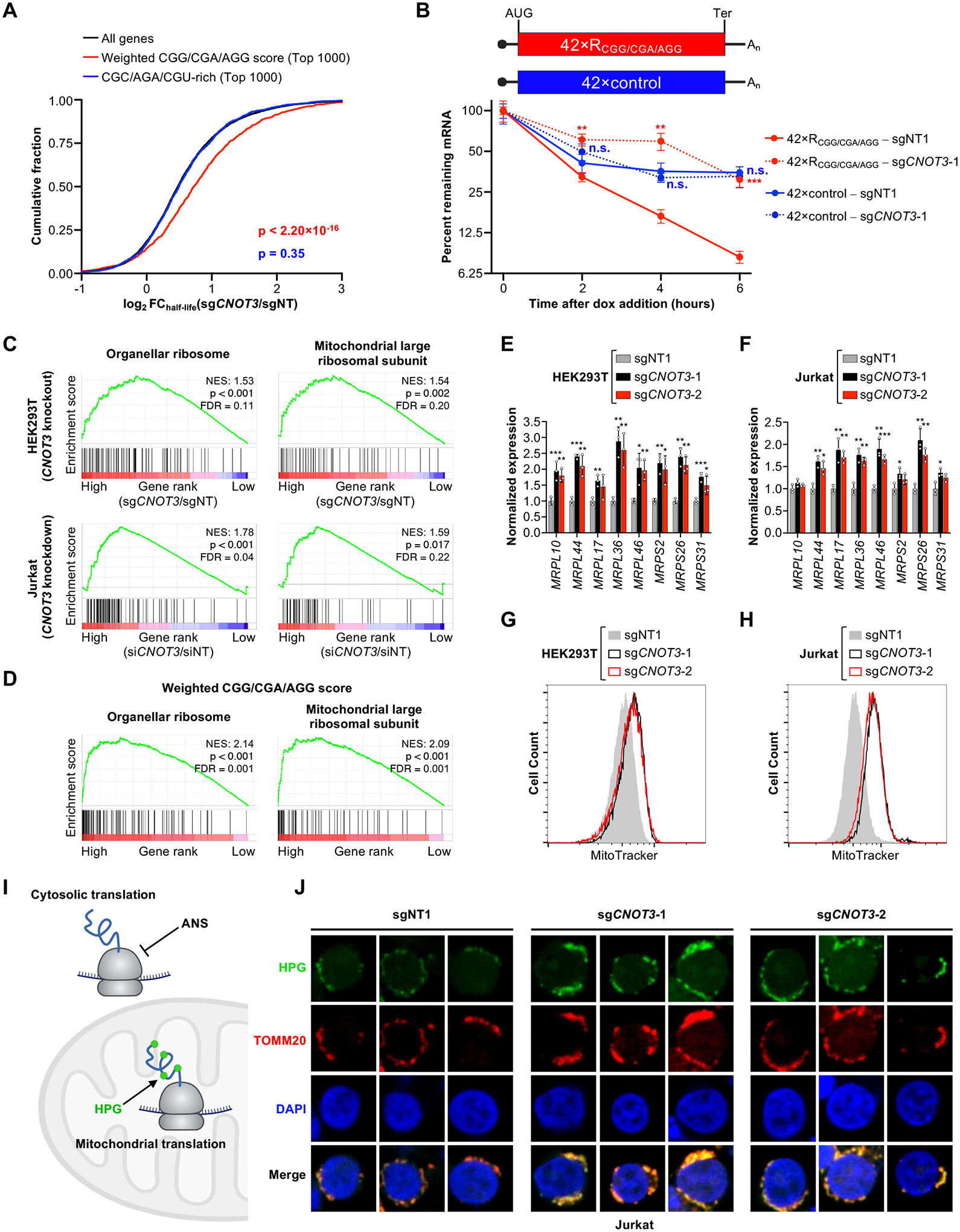
mRNAs rich in CGG, CGA, and AGG arginine codons are destabilized by CNOT3. (**A**) Cumulative distribution function (CDF) plots showing the fold-change in half-lives of the following sets of mRNAs in CNOT3-depleted HEK293T cells, measured by SLAM-seq: i) mRNAs with high weighted CGG/CGA/AGG scores, calculated as the sum of the enrichment values of each of these codons in the P-site of CNOT3-bound ribosomes, normalized to total codon number, in each mRNA; ii) mRNAs rich in arginine encoded by CGC, AGA, and CGU. mRNAs rich in these codons that also had a high weighted CGG/CGA/AGG score (top 1000) were excluded from this gene set. *P* values were calculated by one-sided Wilcoxon rank sum test. (**B**) Stability of a reporter construct encoding 42 arginine-centered tripeptides, with arginine encoded by CGG, CGA, or AGG, or a control reporter with arginine codons replaced with codons that were not enriched in the P-site of CNOT3-bound ribosomes. *CNOT3* knockout (sg*CNOT3*-1) or control (sgNT1) HEK293T cells were treated with 1 μg/mL doxycycline, and reporter mRNA levels relative to *GAPDH* at each time point were measured by qRT-PCR. n=3 biological replicates (mean ± SD shown). *P* values were calculated by student’s t test, comparing sg*CNOT3*-1 to sgNT1 for each reporter. **P<0.01; ***P<0.001; n.s., not significant. (**C**) GSEA analysis of global mRNA half-life data showing stabilization of mRNAs encoding mitochondrial ribosomal proteins in CNOT3-depleted HEK293T cells and Jurkat cells. Genes are ordered left to right along the x-axes based on fold-change of half-lives in CNOT3-depleted cells compared to control cells (high to low). The rank of genes within each geneset is indicated with vertical black lines. mRNA stability in CNOT3-depleted Jurkat cells was reported previously (33). (**D**) GSEA showing that human mRNAs encoding mitochondrial ribosomal proteins exhibit a high weighted CGG/CGA/AGG score. Genes are ordered left to right along the x-axes based on weighted CGG/CGA/AGG score (high to low). The rank of genes within each geneset is indicated with vertical black lines. (**E** and **F**) qRT-PCR analysis of mitochondrial ribosomal protein mRNAs, normalized to *GAPDH*, in HEK293T (E) and Jurkat cells (F) infected with lentivirus expressing the indicated sgRNAs. n=3 biological replicates (mean ± SD shown). *P* values were calculated by student’s t test. *P<0.05; **P<0.01; ***P<0.001. (**G** and **H**) Flow cytometry analysis of HEK293T (G) and Jurkat cells (H) expressing the indicated sgRNAs and stained with MitoTracker. Representative data from n=3 biological replicates shown. (**I**) Schematic of mitochondrial translation assay. Cytosolic translation was inhibited with anisomycin (ANS) and nascent mitochondrial peptides were labeled with L-homopropargylglycine (HPG). Figure created with BioRender.com. (**J**) Representative images of HPG-labeled Jurkat cells expressing the indicated sgRNAs. TOMM20 is a mitochondrial membrane protein. Representative data from n=3 biological replicates shown.

To examine the relationship between codon enrichment in CNOT3-bound ribosomes and mRNA decay, we used SLAM-seq (31) to globally measure mRNA half-lives in control and CNOT3-deficient HEK293T cells (fig. S2A). As reported (32), loss of CNOT3 resulted in reduced fitness (fig. S2B). Because P-site CGG, CGA, and AGG arginine codons were most strongly associated with recruitment of CNOT3 to translating ribosomes, we stratified mRNAs by calculating a weighted CGG/CGA/AGG score. Each of these codons was assigned a value equal to its enrichment in the P-site of CNOT3-bound ribosomes (Fig. 1F). The score for each mRNA was defined as the sum of the weighted values of these codons, normalized to the total number of codons, in the ORF. Transcripts with high weighted CGG/CGA/AGG scores were preferentially stabilized upon loss of CNOT3 (Fig. 2A). Transcripts rich in other arginine codons (CGC, AGA, and CGU) did not exhibit this behavior (Fig. 2A). Analysis of mRNA decay rates in CNOT3-depleted Jurkat cells (33) or steady-state mRNA levels in pro-B cells from *Cnot3* knockout mice (34) further confirmed that transcripts rich in CGG, CGA, or AGG arginine codons, but not arginine encoded by CGC, AGA, or CGU, were preferentially stabilized in CNOT3-deficient cells (fig. S2, C and D).

To further assess whether select arginine codons are sufficient to promote CNOT3-mediated mRNA decay, we constructed a doxycycline-regulated reporter transcript encoding 42 tripeptides, each centered on a CGG, CGA, or AGG arginine codon (Fig. 2B). A control mRNA, with each arginine codon replaced with a codon that was not enriched in the P-site of CNOT3-bound ribosomes, was also generated. Consistent with our transcriptome-wide analyses, the arginine-encoding reporter decayed significantly faster than the control reporter and was selectively stabilized upon CNOT3 depletion. Together, these data provide strong evidence that CNOT3 recruitment to translating ribosomes by CGG, CGA, and AGG arginine codons results in accelerated mRNA degradation.

## Mitochondrial ribosomal protein mRNAs are enriched in CGG/CGA/AGG codons and regulated by CNOT3

We next investigated the endogenous mRNAs that are most strongly regulated by CNOT3 due to the presence of destabilizing arginine codons. Gene set enrichment analysis (GSEA) (35) of mRNA decay rate data from HEK293T and Jurkat cells, as well as steady-state mRNA levels from *Cnot3* knockout pro-B cells, revealed that genesets containing mitochondrial ribosomal proteins were highly upregulated upon CNOT3 depletion and were the only significantly upregulated genesets detected in all three datasets (Fig. 2C and fig. S2, E to G). To determine whether these effects were related to an enrichment of destabilizing arginine codons, we ranked genes according to their weighted CGG/CGA/AGG scores. Indeed, genesets containing mitochondrial ribosomal proteins were the most highly enriched genesets according to this metric (Fig. 2D and fig. S2H).

These findings indicate that mitochondrial ribosomal proteins are regulated by CNOT3. We confirmed that depletion of CNOT3 in HEK293T or Jurkat cells resulted in a strong increase in the steady-state abundance of these transcripts (Fig. 2, E and F) and a corresponding increase in mitochondrial mass (Fig. 2, G and H). Furthermore, fluorescent labeling of nascent mitochondrial peptides demonstrated an increase in mitochondrial translation (Fig. 2, I and J). Depletion of CNOT1, the scaffolding subunit of the CCR4-NOT complex, resulted in a similar increase in the steady-state abundance of mitochondrial ribosomal protein mRNAs (fig. S2, I and J). Thus, regulation of mitochondrial ribosomal proteins, which are rich in arginines encoded by CGG, CGA, and AGG codons, by CCR4-NOT impacts mitochondrial homeostasis in mammalian cells.

## Structural analysis of co-translational CNOT3 recruitment by P-site arginine codons

**Fig. 3.**
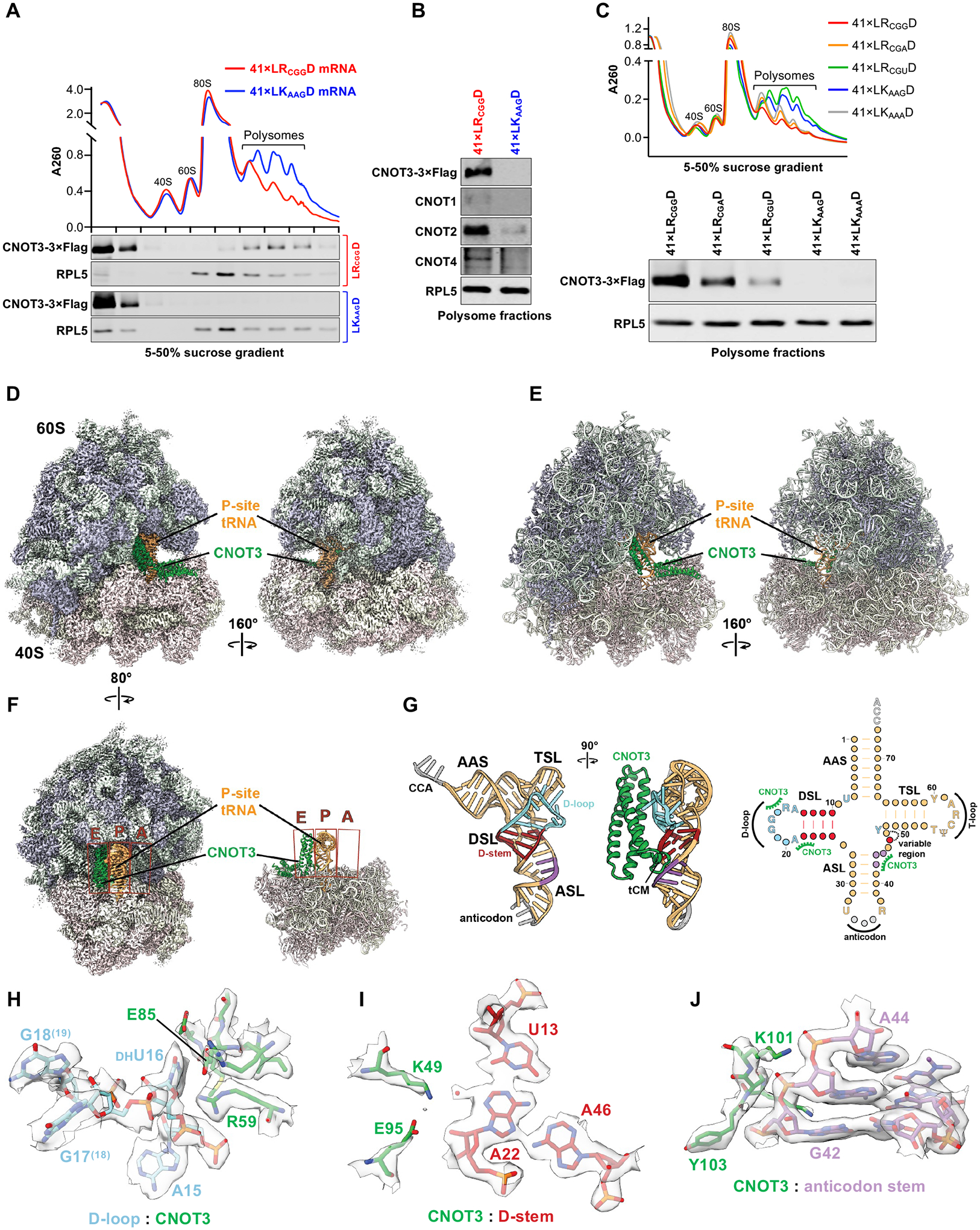
Structural analysis of a human CNOT3-ribosome complex. (**A**) Sucrose density gradient profiles and western blot analysis of *in vitro* translation reactions performed with 41×LR_CGG_D or 41×LK_AAG_D mRNA. (**B**) Western blot analysis of CCR4-NOT components in combined polysome fractions from *in vitro* translation reactions assembled as in (A). (**C**) Sucrose density gradient profiles and western blot analysis of combined polysome fractions from *in vitro* translation reactions assembled on the indicated mRNAs. Representative data from n=3 biological replicates shown for panels A-C. (**D** and **E**) Cryo-EM density map (D) and cartoon model (E) of the human CNOT3-ribosome complex. The 60S subunit is shown in cyan/grey and the 40S subunit in light blue/grey. CNOT3 is highlighted in green and the P-site tRNA in orange. (**F**) Clipped density map (left) and atomic model (right) highlighting the ribosomal E, P, and A-sites. CNOT3 and the P-site tRNA are colored as before. (**G**) Cartoon model and secondary structure representation of CNOT3-arginyl tRNA interactions, highlighting tRNA elements in the D-loop (cyan), D-stem (red), and anticodon stem (purple) contacted by CNOT3 (green). AAS, amino acid acceptor stem; TSL, T stem loop; ASL, anticodon stem loop; DSL, D stem loop. (**H-J**) Experimental Cryo-EM density (grey surface) of the three CNOT3/tRNA interaction elements: D-loop (H), D-stem (I), and anticodon stem (J). Modeled residues are shown as sticks and colored as in panel G.

To further corroborate our finding that select P-site arginine codons stimulate co-translational CNOT3 recruitment and to investigate the underlying mechanism, we established an efficient human *in vitro* translation system from HEK293T lysates. Endogenous mRNAs were removed by micrococcal nuclease, enabling assembly of polysomes on exogenously added mRNAs of any desired sequence (fig. S3A). To determine whether co-translational CNOT3 recruitment was recapitulated in this system, we constructed an mRNA encoding 41 repeats of leucine-arginine-aspartic acid (LRD), the most enriched tripeptide in CNOT3-bound ribosomes (fig. S1O), with arginine encoded by CGG (41×LR_CGG_D). As a control, the arginine codon was replaced with a lysine codon (41×LK_AAG_D). As predicted from our selective ribosome profiling data, *in vitro* translation of 41×LR_CGG_D, but not 41×LK_AAG_D, resulted in robust recruitment of Flag-tagged CNOT3 to polysomes (Fig. 3A). Other CCR4-NOT complex components were also selectively recruited to the 41×LR_CGG_D mRNA (Fig. 3B). Moreover, the hierarchy of P-site arginine codon-mediated recruitment of CNOT3 was recapitulated in this system, as 41×LR_CGG_D recruited the most CNOT3 to polysomes, followed by 41×LR_CGA_D and 41×LR_CGU_D, while no detectable CNOT3 association was observed upon translation of 41×LK_AAG_D and 41×LK_AAA_D mRNAs (Fig. 3C). Translation of an additional arginine-rich mRNA containing 73 CGG, CGA, or AGG codons also stimulated recruitment of CNOT3 to polysomes, while replacement of the arginine codons with codons that were not enriched in the P-site of CNOT3-bound ribosomes abolished recruitment (fig. S3B).

This system was then leveraged to determine the structural basis of CNOT3 recruitment to ribosomes with P-site arginine codons. Polysomes actively translating 41×LR_CGG_D mRNA transcripts were enriched for CNOT3-bound ribosomes by Flag IP and analyzed by cryo-EM (fig. S3C). Micrographs of these samples showed linearly clustered ribosomes, indicating that polysomes remained intact during sample preparation (fig. S4A). We analyzed individual ribosomes (but not disomes or higher order polysomes) for structure determination. The single particle reconstruction of these ribosomes yielded a homogeneous structure with an overall resolution of 2Å (Fig. 3D, fig. S4, and table S1). Density interpretation and model building revealed an empty A-site, a tRNA in the P-site, and CNOT3 occupying the E-site (Fig 3, E and F). As observed in previous yeast and rabbit structures (14, 19), the N-terminal 3-helix bundle (aa 1–111) of CNOT3 bridges the two ribosomal subunits and contacts the D-loop, D-stem, and anticodon stem of the P-site tRNA (Fig. 3, F to J, and fig. S5, A to C). An additional helical domain of CNOT3, composed of amino acids 112–231, contacts uS7 and eS25, and extends out of the E-site. The C-terminal portion of CNOT3, which mediates interactions with other CNOT components and is connected to the N-terminal modules via a disordered linker, is not visible in our reconstruction. The codon-anticodon pairing (fig. S5D and fig. S6, A and B) and defined CCA-RLD density connecting the P-site tRNA and the nascent peptide chain (fig. S5E and fig. S6, A and C) unambiguously identified the arginine-encoding CGG codon in the P-site, as predicted from our selective ribosome profiling data. The CGG codon present in our transcript can be recognized by the two tRNA^Arg,CCG^ isodecoders or, due to wobble base-pairing, by one of the five tRNA^Arg,UCG^ isodecoders present in HEK293T cells (36). Northern blot analysis revealed that all of these tRNAs are present in the CNOT3-enriched sample (fig. S6, D and E). For model building, we chose the most abundant isodecoder, tRNA^Arg,CCG−1^, including its post-transcriptional modifications, which are clearly identifiable in our reconstruction (fig. S6F). This structural model provided an opportunity to investigate the molecular interactions that promote CNOT3 recruitment when select arginine codons occupy the ribosomal P-site.

## The P-site tRNA D-arm is a key determinant of co-translational CNOT3 recruitment

It has been reported that select codon pairs in the ribosomal P- and A-sites can distort the A-site mRNA configuration, resulting in impaired decoding (37). This could potentially lead to CNOT3 recruitment as a consequence of ribosomal stalling. Unlike previous reconstructions of Not5 and CNOT3-bound ribosomes, we were able to model the A-site mRNA. We therefore compared the mRNA geometry in our structure to the structure of mRNA in a translating ribosome with a tRNA probing the A-site (38) (fig. S7A). The conformation of the A-site mRNA, as well as the overall structure of CNOT3-bound ribosomes, were compatible with decoding, suggesting that recruitment of CNOT3 was not a consequence of ribosomal stalling due to mRNA distortion.

We next considered the possibility that the specific interactions between the N-terminal domain of CNOT3 and arginyl-tRNA visualized in our structure play a role in CNOT3 recruitment. The second and third N-terminal helices of CNOT3 contact the arginyl-tRNA D-loop and D-stem, while a short element named the tRNA clamp motif (tCM) (14) interacts with the tRNA anticodon stem (Fig. 3, G to J, and fig. S5, A to C). We hypothesized that sequence and structural differences among tRNAs could impact these interactions and thereby influence CNOT3 recruitment when distinct codons, and consequently distinct tRNAs, occupy the P-site.

**Fig. 4.**
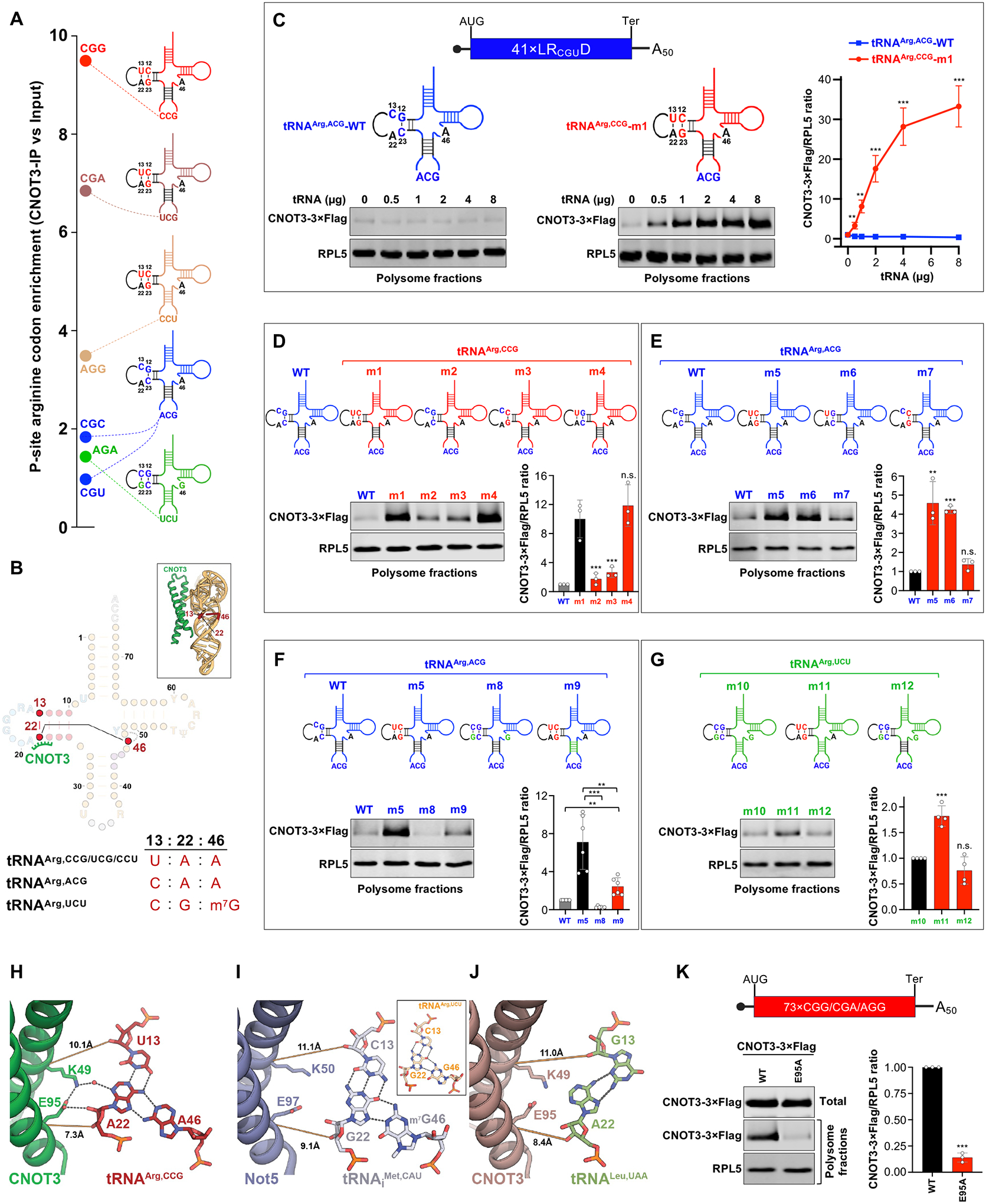
A U13:A22:A46 triplet in select arginine tRNAs promotes co-translational CNOT3 recruitment. (**A**) Schematic of arginine tRNAs, showing their key distinguishing features and the codons they decode, arranged by their enrichment in the P-site of CNOT3-bound ribosomes. (**B**) Secondary structure and cartoon depictions of tRNA and CNOT3, highlighting the CNOT3 interaction with nucleotide 22 of the P-site tRNA as well as the 13:22:46 base triplet. (**C** to **G**) *In vitro* translation of 41×LR_CGU_D mRNA in the presence of *in vitro* transcribed arginine tRNA variants, followed by western blot analysis of combined polysome fractions to assess CNOT3 recruitment. For panels D-G, 2 μg tRNA per 100 μL reaction volume were used. All experiments were performed with n=3–6 biological replicates (mean ± SD shown). *P* values were calculated by student’s t test, comparing to m1 (D), WT (E), m10 (G), or as indicated with brackets (F). **P<0.01; ***P<0.001; n.s., not significant. (**H** to **J**) Structural models of CNOT3/Not5 interactions with the 13:22:46 base triplet of tRNA^Arg,CCG^ (H), tRNA_i_^Met^ from PDB 6TB3 (14) (I), and the rebuilt tRNA^Leu,UAA^ model (PDB 93CI) (19) (J). The inset in (I) shows the tRNA^Arg,UCU^ 13:22:46 triplet from PDB 8ISS (45). (**K**) *In vitro* translation of the 73×CGG/CGA/AGG mRNA in lysates from cells expressing Flag-tagged wild-type CNOT3 or CNOT3 E95A, followed by western blot analysis of combined polysome fractions. n=3 biological replicates (mean ± SD shown). *P* values were calculated by student’s t test. ***P<0.001.

The selective association of CGG, CGA, and AGG arginine codons with CNOT3 recruitment, in contrast to CGC, AGA, and CGU arginine codons, provides a framework for investigating tRNA sequence features that may impact CNOT3 binding. In human cells, the six arginine codons are decoded by five distinct isoacceptor tRNA families (Fig. 4A and fig. S7B). The D-loops of all arginine tRNAs are identical due to their essential role in recognition by arginyl tRNA synthetase (RARS) (39, 40). tRNA^Arg,CCG^ and tRNA^Arg,UCG^ decode the most enriched P-site codons in CNOT3-bound ribosomes (CGG and CGA) and, in addition to the conserved D-loop, also share identical D-stem and anticodon stem sequences (Fig. 4A and fig. S7B). The third most enriched arginine codon (AGG) is decoded by tRNA^Arg,CCU^, which shares the D-arm sequence with tRNA^Arg,CCG/UCG^, but possesses a distinct anticodon stem. The least enriched arginine codons (CGC, AGA, and CGU) are decoded by tRNA^Arg,ACG^ and tRNA^Arg,UCU^, which have different D-stems compared to tRNA^Arg,CCG/UCG/CCU^. Most notably, the D-stems of tRNA^Arg,CCG/UCG/CCU^, tRNA^Arg,ACG^, and tRNA^Arg,UCU^ engage in distinct triplet base interactions involving tRNA positions 13, 22 and 46 (Fig. 4B). These observations suggest that variations in the D- and anticodon stems of arginine tRNAs may alter their affinity for CNOT3 in the context of a translating ribosome. They also raised the possibility that nucleotide modifications, particularly the absence of m^7^G at nucleotide 46 in tRNA^Arg,CCG/UCG/CCU^, which participates in the aforementioned D-stem triplet base interaction, could impact CNOT3 recruitment.

To examine these possibilities, we used *in vitro* transcription to generate a panel of arginine tRNA mutants*. In vitro* transcribed tRNAs, which lack nucleotide modifications, can efficiently participate in translation when introduced into cells or cell-free translation reactions (41–43), providing an efficient system for assessing the role of sequence features and modifications in CNOT3 recruitment. *In vitro* aminoacylation assays confirmed that none of the mutations impaired tRNA charging compared to each respective parental tRNA (fig. S8A). *In vitro* translation assays were then performed using the 41×LR_CGU_D mRNA, which is decoded by tRNA^Arg,ACG^ and weakly recruits CNOT3 (Fig. 3C). As expected, addition of excess tRNA^Arg,ACG−1^ to these reactions had no effect on CNOT3 recruitment (Fig. 4C and fig. S8B). In contrast, addition of a variant of tRNA^Arg,CCG−1^, reprogrammed to decode the CGU codon by mutating the anticodon to ACG (tRNA^Arg,CCG^-m1), promoted CNOT3 recruitment in a dose-dependent manner (Fig. 4C and fig. S8C). These data demonstrate that the sequence of the P-site tRNA plays a key role in co-translational CNOT3 recruitment and show that this effect is not dependent on specific tRNA nucleotide modifications.

We next tested whether the distinct D-stem sequences of tRNA^Arg,ACG^ and tRNA^Arg,CCG^ were responsible for the contrasting abilities of these tRNAs to recruit CNOT3. These D-stems differ by three nucleotides (Fig. 4C, C13 vs. U13; base-pair G12:C23 vs. C12:G23). Swapping the D-arm of tRNA^Arg,CCG^-m1 (tRNA^Arg,CCG^ reprogrammed to decode the CGU arginine codon) with the D-arm of tRNA^Arg,ACG^ (generating tRNA^Arg,CCG^-m2) abrogated CNOT3 recruitment to the 41×LR_CGU_D mRNA (Fig. 4D and fig. S8D, compare m1 to m2). Moreover, mutation of U13 to C13 (tRNA^Arg,CCG^-m3) was sufficient to impair CNOT3 recruitment, while reversing the C12:G23 to a G12:C23 base-pair had no effect (tRNA^Arg,CCG^-m4). We further showed that addition of the D-stem sequence from tRNA^Arg,CCG^ to tRNA^Arg,ACG^ (tRNA^Arg,ACG^-m5), or the single C13U mutation enabling formation of the U13:A22 base pair in tRNA^Arg,ACG^ (tRNA^Arg,ACG^-m6) was sufficient to enable CNOT3 recruitment (Fig. 4E and fig. S8E, compare WT to m5 and m6). Again, flipping the G12:C23 base-pair (tRNA^Arg,ACG^-m7) had no effect in this context. Altogether, these results pinpoint position U13 in the tRNA^Arg,CCG/UCG/CCU^ D-arm, which forms a triplet base interaction with A22 and A46 (Fig. 4B), as a critical feature associated with CNOT3 recruitment to ribosomes.

In tRNA^Arg,UCU^, the triplet interaction is C13:G22:m^7^G46 (Fig. 4B), a frequent configuration of nucleotides at these positions among all tRNAs (fig. S9), whereas tRNA^Arg,ACG^ has an unusual C13:A22:A46 triplet. To further probe how this triplet base interaction influences CNOT3 recruitment, we mutated tRNA^Arg,ACG^ at positions 22 and 46 to introduce the common C13:G22:G46 triplet (tRNA^Arg,ACG^-m8). These mutations did not increase CNOT3 recruitment (Fig. 4F and fig. S8F, compare WT to m8). Thus, the U13:A22:A46 triplet, present in tRNA^Arg,CCG/UCG/CCU^, appears to be optimally configured to recruit CNOT3.

To determine why the U13:A22:A46 triplet favors CNOT3 recruitment, we further examined our structural model. Nucleotide A22 makes direct and water-mediated H-bonding interactions with CNOT3 residues E95 and K49, respectively (Fig. 4H). Supporting the importance of these interactions, an E95A substitution in CNOT3 greatly reduced co-translational recruitment to a CGG/CGA/AGG-rich transcript (Fig. 4K and fig. S8J). In the previously reported structures of Not5/CNOT3-bound ribosomes, yeast tRNA_i_^Met^ or rabbit tRNA^Leu,UAA^, which lack the U13:A22:A46 triplet, occupy the P-site (14, 19). We therefore examined the Not5/CNOT3-tRNA interaction interfaces in these structures to determine how alternative 13:22:46 triplet configurations affect Not5/CNOT3 binding. tRNA_i_^Met^ has the most common triplet combination (C13:G22:m^7^G46), while tRNA^Leu,UAA^ has a *trans*-Hoogsteen G13:A22 pairing characteristic of type-II tRNAs (44). The distinct base configurations in tRNA_i_^Met^ and tRNA^Leu,UAA^ increase the intermolecular distance to Not5 and CNOT3 in their respective complexes. This prevents the formation of the hydrogen-bonding network observed in our CNOT3/tRNA^Arg,CCG^ structure (Fig. 4, I and J). Because the C13:G22:m^7^G46 triplet of tRNA^Arg,UCU^, visualized in a structure of this tRNA with a splicing endonuclease (45), is structurally similar to tRNA_i_^Met^ (Fig. 4I, inset), we expect that interactions between tRNA^Arg,UCU^ and CNOT3 are similarly disrupted. These observations provide a structural basis for the enhanced recruitment of CNOT3 by arginine tRNAs containing the U13:A22:A46 triplet base interaction.

## Impact of the P-site tRNA anticodon stem on co-translational CNOT3 recruitment

Our CNOT3-ribosome structure, as well as previously reported structures (14, 19), also revealed interactions between CNOT3 and the anticodon stem of the P-site tRNA. These interactions are mediated by the tCM, which forms several direct backbone interactions as well as water-bridged interactions with bases G42 and A43 of the P-site tRNA (fig. S8H). Supporting the importance of these interactions, a K105S substitution in the tCM abrogated recruitment of CNOT3 to a CGG/CGA/AGG-rich transcript (fig. S8, I and J). We hypothesized that these interactions might explain why tRNA^Arg,CCG^ and tRNA^Arg,UCG^, which share an anticodon stem, recruit more CNOT3 compared to tRNA^Arg,CCU^, which has a distinct anticodon stem but the same D-stem and D-loop structure (Fig. 4A and fig. S7B). The unique anticodon stem of tRNA^Arg,UCU^ may also contribute to the poor CNOT3 recruitment associated with this tRNA. To test whether the anticodon stem contributes to differential CNOT3 recruitment by these tRNAs, we replaced the anticodon stem of tRNA^Arg,ACG^ bearing the tRNA^Arg,CCG^ D-arm (Fig. 4F, tRNA^Arg,ACG^-m5) with the anticodon stem from tRNA^Arg,UCU^ (Fig. 4F, tRNA^Arg,ACG^-m9). Consistent with our hypothesis, this greatly reduced, but did not eliminate, CNOT3 binding (Fig. 4F and fig. S8F, compare m5 to m9). In addition, we reprogrammed tRNA^Arg,UCU−1^ to recognize the CGU arginine codon (Fig. 4G, tRNA^Arg,UCU^-m10), which showed that addition of the tRNA^Arg,CCG^ U13:A22:A46 triplet (Fig. 4G, tRNA^Arg,UCU^-m11), but not the tRNA^Arg,CCG^ anticodon stem (Fig. 4G, tRNA^Arg,UCU^-m12), was sufficient to increase CNOT3 recruitment (Fig. 4G and fig. S8G). Thus, the presence of the U13:A22:A46 triplet is the major determinant of CNOT3 recruitment by arginine tRNAs, while the anticodon stems play a lesser, but measurable, role. Although we lack the structural information to precisely determine how sequence variation in the anticodon stem impacts CNOT3 interactions, we suspect that different base-pairing configurations may alter the tCM-tRNA affinity. In keeping with this concept, the anticodon stems of tRNA^Arg,UCU^ and tRNA^Arg,CCU^ both contain G:U wobble base pairs, which can locally alter the twist of the RNA backbone (fig. S8K) (46), potentially affecting interactions with the CNOT3 tCM element.

## An extra nucleotide upstream of the D-loop GG motif prevents CNOT3 recruitment

**Fig. 5.**
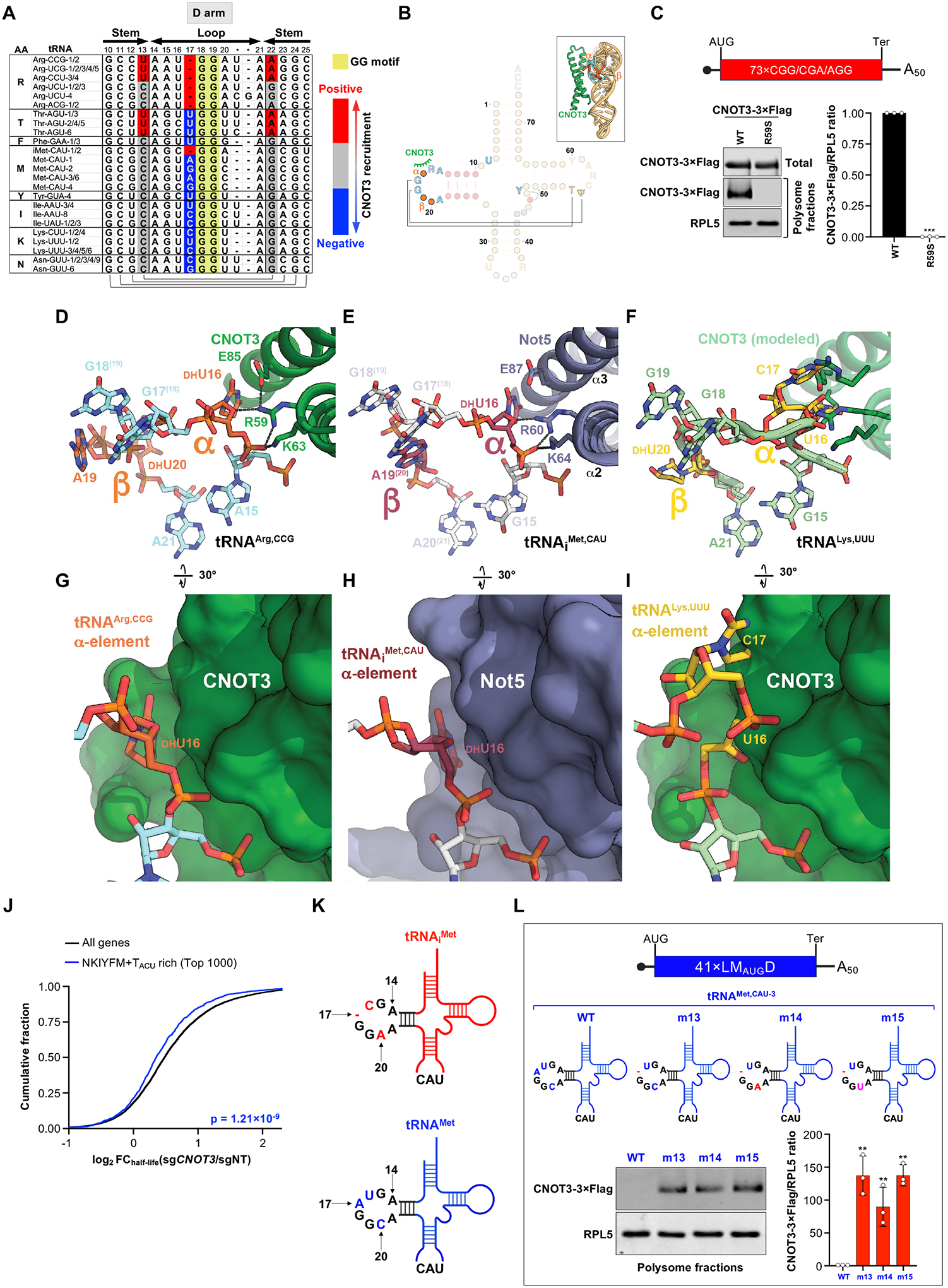
An extra nucleotide in the D-loop α element preceding the GG motif blocks CNOT3 recruitment. (**A**) Alignment of the D-arms of human arginine tRNAs and tRNAs that have an extra nucleotide in the α element. (**B**) Secondary structure and cartoon depictions of tRNA and CNOT3, highlighting the CNOT3 interaction with the D-loop α element of the P-site tRNA. (**C**) *In vitro* translation of the 73×CGG/CGA/AGG mRNA in lysates from cells expressing Flag-tagged wild-type CNOT3 or CNOT3 R59S, followed by western blot analysis of combined polysome fractions. n=3 biological replicates (mean ± SD shown). *P* values were calculated by student’s t test. ***P<0.001. (**D** to **I**) Molecular models of CNOT3/Not5 interactions with the α element of P-site tRNA^Arg,CGG^ (D and G), tRNA_i_^Met^ from PDB 6TB3 (14) (E and H) and tRNA^Lys,UUU^ from PDB 6SGC (48) with an aligned, superposed CNOT3 model from tRNA^Arg,CGG^ (F and I). (**J**) CDF plot showing the fold-change in half-lives of mRNAs rich in codons decoded by tRNAs with the α element insertion relative to other transcripts in CNOT3-depleted HEK293T cells, measured by SLAM-seq. *P* value calculated by one-sided Wilcoxon rank sum test. (**K**) Comparison of the D-arms of human tRNA_i_^Met^ and tRNA^Met^. (**L**) *In vitro* translation of 41×LM_AUG_D mRNA in the presence of *in vitro* transcribed tRNA^Met^ variants (10 μg tRNA per 100 μL reaction volume), followed by western blot analysis of combined polysome fractions to assess CNOT3 recruitment. All experiments were performed with n=3 biological replicates (mean ± SD shown). *P* values were calculated by student’s t test, comparing mutants to WT. **P<0.01.

Our structural studies revealed that select arginine tRNAs possess distinct sequence and structural features that promote CNOT3 recruitment. To determine whether other tRNAs have features that inhibit recruitment of CNOT3, we examined the codons that were most depleted from the P-site of CNOT3-bound ribosomes (fig. S10A). We noticed that many of the most depleted codons, including codons for asparagine (N), lysine (K), isoleucine (I), tyrosine (Y), methionine (M), phenylalanine (F), and threonine (T), are decoded by tRNAs with an extra nucleotide in the α element of the D-loop, immediately preceding the universally conserved GG D-loop motif (Fig. 5, A and B, and fig. S9) (44). The GG motif forms a key interaction with the T-arm to stabilize the “elbow” structure of all tRNAs. α and β D-loop elements flank the GG motif and often contain modified dihydrouridine nucleotides (47). In our structure, CNOT3 contacts the α element of the D-loop, but not the β element (Fig. 5, B, D, and G), mirroring the interface between Not5/tRNA_i_^Met^ in yeast and CNOT3/tRNA^Leu,UAA^ in rabbit reticulocyte lysate (Fig. 5, E and H, and data not shown) (14, 19). This interaction is critical for CNOT3 recruitment, as demonstrated by mutating the key CNOT3 residue (R59) that contacts this site in the tRNA (Fig. 5C and fig. S10B). A structural overlay of a tRNA with an extra nucleotide in the α element (tRNA^Lys,UUU^) bound in the P-site (48) shows that the α expansion creates a steric clash with CNOT3 (Fig. 5, F and I). The same D-loop orientation is also observed in the crystal structure of isolated bovine tRNA^Lys,UUU^ (49), suggesting that it represents a structurally rigid bulge within the α-element (fig. S10C). These observations suggest that depletion of select P-site codons may result from a steric clash between CNOT3 and the cognate tRNAs for these amino acids that prevents effective CNOT3 binding in the E-site. Consistent with this hypothesis, we observed that mRNAs rich in N, K, I, Y, M, F, and T codons decoded by tRNAs with an extra nucleotide in the α element were less sensitive to CNOT3-mediated decay compared to other transcripts (Fig. 5J and fig. S10, D and E). Notably, cytosolic ribosomal proteins represent the class of transcripts most enriched for these codons (fig. S10, F and G), providing a possible explanation for our finding that depletion of CNOT3 selectively upregulates mitochondrial, but not cytosolic, ribosomal proteins.

To directly test the effect of the α element insertion on CNOT3 recruitment, we performed further tRNA mutagenesis experiments. We noted that, among tRNAs that decode methionine codons, the initiator tRNA_i_^Met^ has a single residue in the α element, while all elongator tRNA^Met^ isodecoders have an additional nucleotide preceding the GG motif (Fig. 5A and fig. S9). A previous structural analysis of Not5-bound ribosomes in yeast identified tRNA_i_^Met^ in the P-site (14). The structural similarity between the Not5/tRNA and CNOT3/tRNA interfaces suggests that CNOT3 recruitment may also be compatible with the presence of a similar tRNA in the P-site. Furthermore, mutations in the D-arm of elongator tRNA^Met^ that remove the extra α element nucleotide are expected to be compatible with tRNA charging and decoding of internal AUG codons (50, 51).

Confirming the inhibitory effect predicted from our structural studies, deletion of the extra α nucleotide upstream of the GG motif in elongator tRNA^Met,CAU−3^ (tRNA^Met^-m13) was sufficient to stimulate recruitment of CNOT3 to ribosomes translating a 41×LM_AUG_D mRNA (Fig. 5, K and L, and fig. S10, H and I, compare WT to m13). Consistent with our structural analyses, changes to the D-loop β element did not impact CNOT3 recruitment, since introducing C20A (tRNA^Met^-m14) or C20U (tRNA^Met^-m15) mutations had no effect on CNOT3 binding (Fig. 5L and fig. S10, H and I, compare m13 to m14 or m15). Taken together, our results demonstrate that a D-stem U13:A20:A46 triplet and a short, single-nucleotide D-loop α element in the P-site tRNA are major determinants of CNOT3 co-translational recruitment, providing a structural basis to explain predominant patterns of P-site codon enrichment and depletion in CNOT3-bound ribosomes.

## Slow decoding enables P-site tRNA-mediated CNOT3 recruitment to ribosomes

**Fig. 6.**
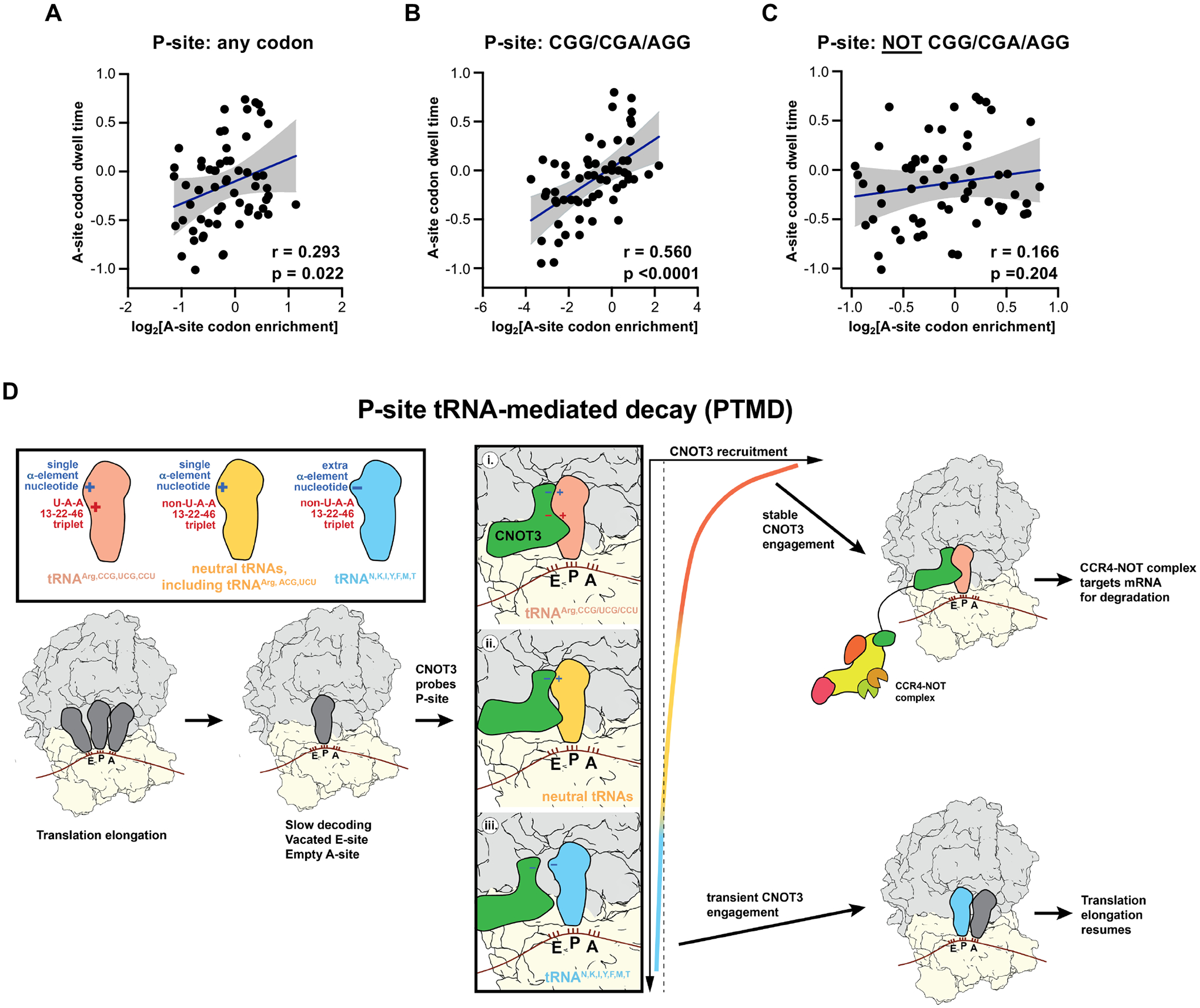
Slow decoding promotes P-site tRNA-mediated decay (PTMD). (**A** to **C**) Pearson correlation of codon enrichment in the ribosomal A-site of CNOT3-bound ribosomes and A-site dwell time in HEK293T cells when the P-site is occupied by any codon (A), a CGG/CGA/AGG codon (B), or any codon other than CGG/CGA/AGG (C). Note that panel A is also shown in Fig. S1N and duplicated here to facilitate comparison with other panels. (**D**) Proposed mechanism of PTMD. Slow decoding, resulting in a ribosome with empty A- and E-sites, provides an opportunity for CNOT3 enter the E-site and probe the P-site tRNA. (i) If the P-site tRNA has the U13:A22:A46 triplet and lacks the extended α-element (i.e., tRNAs that decode CGG/CGA/AGG arginine codons), CNOT3 binding will be stabilized and mRNA decay will be favored. (ii) If the P-site tRNA is neutral, lacking both the extended D-loop α element and the U13:A22:A46 triplet, CNOT3 binding may be transient. However, if an extended ribosomal stall occurs due to scarcity of a charged tRNA that can enter the A-site, CCR4-NOT-mediated decay may occur. (iii) If the P-site tRNA has the extended D-loop α element (e.g., tRNAs that decode N, K, I, Y, M, F, and T), CNOT3 accommodation will be sterically blocked, CNOT3 will exit, and translation will resume.

Notably, we observed that the A-site of human CNOT3-bound ribosomes was not occupied by tRNA (Fig. 3F). This configuration, indicative of slow decoding, was similar to the structure of Not5-bound ribosomes in yeast and CNOT3-bound rabbit ribosomes stalled on a highly non-optimal codon (14, 19). As discussed above, we detected a weak, but statistically significant, correlation between A-site dwell time and CNOT3 recruitment in our selective ribosome profiling data (fig. S1N), indicating that slow decoding measurably promotes CNOT3 recruitment in human cells. We found that the correlation between A-site dwell time and CNOT3 association was greatly enhanced when the P-site was occupied by a CGG, CGA, or AGG arginine codon (Fig. 6, A and B). In contrast, when any other codon was present in the P-site, the dwell time correlation was undetectable (Fig. 6C). These data suggest that, like in yeast and rabbit reticulocyte lysate, slow decoding increases the probability that the ribosomal A- and E-sites will be simultaneously vacant, providing an opportunity for CNOT3 to enter the E-site. Our results demonstrate that CNOT3 subsequently probes the D-arm of the P-site tRNA, which ultimately determines whether CNOT3 stably associates with the ribosome and initiates mRNA decay.

## Discussion

The CCR4-NOT complex can associate with translating ribosomes to accelerate mRNA turnover. A major trigger for CCR4-NOT recruitment to ribosomes is slow decoding, which is sensed by entry of the N-terminal domain of the CNOT3/Not5 subunit into a vacated E-site when the A-site codon is non-optimal and therefore lacks a cognate tRNA. Here we present our finding that, in mammalian cells, the identity of the P-site tRNA plays a critical role in determining how efficiently CNOT3 is recruited to ribosomes. Based on our results, we propose the following model for co-translational CNOT3 recruitment in mammalian cells (Fig. 6D). As in yeast and rabbit reticulocyte lysate, slow decoding, resulting in a ribosomal conformation with empty A- and E-sites, appears to be a pre-requisite for CNOT3 entry into the E-site, enabling it to probe the P-site tRNA. At this stage, three outcomes are possible: (i) If the P-site tRNA has the U13:A22:A46 triplet and lacks the extended α-element (i.e., tRNAs that decode CGG/CGA/AGG arginine codons), CNOT3 binding will be stabilized by direct hydrogen bonding interactions with the P-site tRNA D-arm, resulting in accelerated mRNA decay. (ii) If the P-site tRNA is neutral, lacking both the U13:A22:A46 triplet and the extended D-loop α element, CNOT3 binding may be transient. However, if an extended ribosomal stall occurs due to scarcity of a charged tRNA that can enter the A-site, CCR4-NOT-mediated decay may ensue. (iii) If the P-site tRNA has the extended D-loop α element (i.e., tRNAs that decode N, K, I, Y, M, F, and T), CNOT3 accommodation will be sterically blocked, thereby favoring CNOT3 disengagement and resumption of translation. Because of the central role played by the P-site tRNA in dictating the outcome of CNOT3 recruitment, we propose the term P-site tRNA-mediated decay (PTMD) to refer to this mechanism of accelerated mRNA degradation.

These findings expand our understanding of how codon content influences post-transcriptional regulation. Prior to this work, the impact of specific codons on rates of co-translational mRNA turnover were largely attributed to their effect on decoding efficiency. The results reported here show that specific codons can also modulate the rate of mRNA decay due to the ability of their cognate tRNAs to promote or inhibit association of the CCR4-NOT complex. Thus, in addition to their canonical role in decoding, tRNAs directly participate in the recruitment of post-transcriptional regulators to translating ribosomes. This discovery raises the intriguing possibility that other regulatory complexes that engage ribosomes may be similarly impacted by tRNA identity. Evocative of this concept, it was recently shown that angiogenin, a ribonuclease with high specificity towards tRNA, binds to ribosomes with empty A sites, which activates its nuclease activity (52). Akin to CNOT3, interaction with the P-site tRNA appears to be important for stabilizing the association of angiogenin with ribosomes, although in this case, the identity of the P-site tRNA does not appear to be a determining factor in ribosome binding.

The presence of the U13:A22:A46 triplet and absence of the extra nucleotide in the α element is a conserved feature of metazoan tRNAs that decode arginine codons CGG, CGA, and AGG (fig. S11), suggesting that PTMD is operative across animal species. Nevertheless, it is less clear whether this mechanism is active in yeast, where codon optimality appears to be the dominant signal for Ccr4-Not recruitment. The high level of sequence and structural conservation between CNOT3 and Not5 (57% amino acid identity and 0.75 Å Cα RMSD), coupled with the presence of a U13:A22:A46 triplet and a short α element in arginine tRNAs that decode AGG and AGA (fig. S12A; tRNA^Arg,CCU/UCU^), raises the possibility of a functional PTMD pathway in yeast. Indeed, codons with cognate tRNAs possessing an extra nucleotide in the D-loop α element were generally depleted from the P-site of Not4-bound ribosomes (fig. S12, B and C). Nevertheless, P-site AGG and AGA arginine codons were only marginally enriched. Instead, non-optimal codons were unexpectedly enriched, not only in the A-site as reported (14), but also to a similar extent in the P- and E-sites, whereas no such enrichment was observed in human cells (fig. S12, D and E). We speculate that these differences in codon enrichment patterns between yeast and humans are a consequence of distinct mechanisms of binding of CCR4-NOT to ribosomes between these species. Specifically, the E3 ubiquitin ligase Not4, which interacts with and ubiquitylates ribosomal protein eS7 (53, 54), is a constitutive subunit of Ccr4-Not in yeast (55, 56) and is required for recruitment of Not5 to ribosomes (14). In contrast, the metazoan Not4 homolog CNOT4 does not stably associate with CCR4-NOT (57, 58) and is dispensable for ribosomal recruitment of CNOT3 (19). These findings suggest that, when Not5 is recruited to the E-site of a slowly decoding ribosome in yeast, the associated Ccr4-Not complex may become tethered to the ribosome via Not4. If Not5 does not remain stably bound within the E-site, the ribosome may continue further cycles of elongation while remaining associated with Ccr4-Not, thereby moving the non-optimal codon to the P- or E-site. Importantly, this mechanism would render Ccr4-Not association with the ribosome less dependent upon the stable accommodation of Not5 within the E-site, and therefore less reliant upon specific Not5:P-site tRNA interactions. In agreement with this hypothesis, deletion of the N-terminal domain of Not5 has been shown to reduce but not eliminate its binding to ribosomes (14). Unlike the behavior of Not5 in yeast, we found that the R59S mutation of human CNOT3 that disrupted interaction with the P-site tRNA abolished its recruitment to ribosomes. Thus, mammalian ribosomes require CNOT3 accommodation within the E-site, which depends upon specific P-site tRNA interactions, to stably recruit CCR4-NOT, enabling the PTMD mechanism to dominate in this setting.

While we still have much to learn about the physiologic role of the PTMD pathway, we observed that mitochondrial ribosomal proteins, whose mRNAs are rich in CGG, CGA, and AGG arginine codons, are strongly regulated by this mechanism across diverse cell types. Cytosolic ribosomal proteins, in contrast, are rich in N, K, I, Y, M, F, and T codons, whose cognate tRNAs oppose CNOT3-mediated decay. As a consequence of this regulation, we observed that CNOT3 is a strong repressor of mitochondrial translation and mitochondrial mass. In keeping with these findings, mice with reduced CNOT3 expression exhibit increased respiration and are resistant to obesity, consistent with an increase in mitochondrial energy expenditure (59). Moreover, expression of CNOT3, but not other subunits of the CCR4-NOT complex, is upregulated in obese mice and downregulated after fasting (59), suggesting that regulation of mitochondrial activity by PTMD affects the physiological response to nutrient availability. Interestingly, in yeast, mitochondrial ribosomal protein mRNAs are rich in non-optimal codons, while cytosolic ribosomal proteins are mostly encoded by mRNAs with high levels of optimal codons (17, 20). Accordingly, loss of the Ccr4-Not complex also increases mitochondrial mass in yeast (60). Thus, regulation of mitochondrial homeostasis by CCR4-NOT appears to be highly conserved, although the underlying mechanism of recruitment of Not5/CNOT3 to target transcripts occurs through distinct mechanisms across species. Further investigation of how the PTMD pathway is deployed across metazoans, and the impact of this mechanism of gene regulation on physiology and disease, are important priorities for future investigation.

## Materials and methods:

### Cell culture

Cell lines were obtained from American Type Culture Collection (ATCC). HEK293T cells were cultured in Dulbeccoʹs Modified Eagleʹs Medium (DMEM) (Invitrogen) supplemented with 10% (v/v) fetal bovine serum (Sigma) and 1×Antibiotic-Antimycotic (Invitrogen). Jurkat cells were cultured in RPMI1640 (Invitrogen) supplemented with 10% (v/v) fetal bovine serum (Sigma) and 1×Antibiotic-Antimycotic (Invitrogen). Cell lines were confirmed to be free of mycoplasma contamination.

### Sucrose gradient fractionation of ribosomes

2×10^7^ HEK293T cells were lysed in 500 μL ice-cold lysis buffer [20 mM Tris-HCl (pH 7.5), 150 mM KCl, 15 mM MgCl_2_, 1 mM DTT, 1% Triton X-100, 200 U/mL RNase inhibitor (RNasin, Promega, N2515), 1×protease inhibitor cocktail (cOmplete, EDTA-free, Roche)]. Lysate was clarified by centrifugation at 16,000 g at 4° C for 10 minutes. 400 μL lysate was loaded onto a 5–50% sucrose gradient containing 20 mM Tris-HCl (pH 7.5), 150 mM KCl, 5 mM MgCl_2_, and 1×EDTA-free Protease Inhibitor Cocktail, followed by centrifugation at 40,000 rpm at 4° C for 2 hours using a TH-641 rotor (ThermoFisher Scientific). For RNase-treated samples, 500 μL lysate containing 200 μg RNA was digested with 2 μg RNase A at room temperature for 15 minutes prior to loading onto the gradient. For sucrose gradient fractionation of *in vitro* translation reactions, 5–50% sucrose gradients containing 20 mM HEPES (pH 7.5), 100 mM KCl, 5 mM MgCl_2_, and 1×EDTA-free protease inhibitor cocktail were used. Samples were fractionated on a Piston Fractionator (BioComp).

### CNOT3-monosome immunoprecipitation

Sucrose gradient fractionation of RNase A-treated HEK293T lysate was performed as described above. The monosome fractions were collected and combined. 10 μg CNOT3 antibody (ProteinTech, 11135–1-AP; RRID: AB_2229682) or M2 Flag antibody (Sigma, F3165; RRID: AB_259529) was coupled with 100 μL DynaBeads protein G (ThermoFisher Scientific, 10003D). The combined monosome fraction was incubated with the antibody-coupled beads at 4° C with slow rotation for 1 hour. Rabbit or mouse normal IgG was used as the IP negative control. Beads were washed 4 times with ice-cold wash buffer [20 mM Tris-HCl (pH 7.5), 150 mM KCl, 15 mM MgCl_2_, 1% Triton-X100, and 1×EDTA-free protease inhibitor], followed by elution with 1×NuPAGE LDS Sample Buffer (ThermoFisher Scientific, NP0008), and analysis by western blotting.

### CNOT3-selective ribosome profiling

Approximately 6×10^7^ HEK293T cells in 15-cm dishes were harvested by scraping, washed with ice-cold PBS, and incubated in 3 mL of lysis buffer [20 mM Tris-HCl (pH 7.5), 150 mM KCl, 15 mM MgCl_2_, 1 mM DTT, 1% Triton-X100, and 1×EDTA-free protease inhibitor] on ice for 15 minutes. Lysate was clarified by centrifugation at 20,000 g at 4° C for 5 minutes and RNA concentration was measured by Nanodrop. 3 mL lysate containing 2 mg total RNA was incubated with 1660 U RNase I (ThermoFisher Scientific, AM2295) at 4° C with gentle shaking for 5 minutes. Digested lysate was loaded on a 10%–35% sucrose gradient containing 20 mM Tris-HCl (pH 7.5), 150 mM KCl, 5 mM MgCl_2_, and 1×EDTA-free Protease Inhibitor Cocktail, and centrifuged in a TH-641 rotor at 40,000 rpm at 4° C for 2 hours. Monosome fractions were collected and combined. 600 U SUPERase•In RNase Inhibitor (ThermoFisher Scientific, AM2694) and 400U RNase inhibitor RNasin (Promega) were added to the monosome fraction. 100 μL of the monosome fraction was saved for isolation of total ribosomal footprints (input), and 3 mL of the monosome fraction was used for CNOT3-IP.

20 μg of CNOT3 antibody (ProteinTech, 11135–1-AP; RRID: AB_2229682) was incubated with the monosome fraction at 4° C for 2 hours with gentle nutation, followed by addition of 200 μL DynaBead-Protein G and incubation at 4° C for 2 hours. Beads were washed 4 times with wash buffer [20 mM Tris-HCl (pH 7.5), 150 mM KCl, 15 mM MgCl_2_, 1% Triton-X100, and 1×EDTA-free protease inhibitor]. During the last wash, resuspended beads were transferred to a new microcentrifuge tube. Ribosomal footprints were isolated using the Direct-zol RNA miniprep cleanup kit (Zymo Research, R2050). The footprint library was generated and sequenced as described previously (61) with the following modifications:

1. Ribosomal footprints were size selected by gel electrophoresis as described previously (62) and fragments between 17–35 nt were excised for library preparation.
2. Samples were not pooled after linker ligation, and the subsequent steps were performed individually for each sample.
3. 3ʹ linker-ligated RNA fragments were purified using gel extraction as previously described (62).
4. Ribosomal RNA was depleted as described previously (62).
5. n=2 biological replicates were performed.

For analysis of sequencing data, only 29 nt, 30 nt, 32 nt, and 35 nt footprints were used due to their superior triplet periodicity relative to reads of other lengths. For 29–30 nt footprints, the 13^th^ nucleotide was assigned as P-site; for 32 nt and 35 nt footprints, the 14^th^ nucleotide was assigned as P-site. To calculate the enrichment of each codon and amino acid in the E-, P-, or A-sites of CNOT3-bound ribosomes, the significantly enriched footprints in CNOT3-IP samples relative to input (Fisher’s exact test; FDR<0.01) were first identified. The percentage of enriched footprints with each codon in the E-, P-, or A-sites, divided by the percentage of input footprints with each codon in the E-, P-, or A-sites, was then determined.

### CRISPR-Cas9 mediated gene knockout

sgRNAs targeting human *CNOT3*, as well as non-target (NT) sgRNAs (sequences provided in Table S2), were cloned into the lentiCRISPR_v2 vector (Addgene #52961). Lentivirus was packaged in HEK293T cells as described previously (63). Two days after lentiviral transduction, cells were selected in medium containing 0.5 μg/mL puromycin for 6 days before analysis.

### Measurement of mRNA half-life by SLAM-seq

SLAMseq Kinetics Kit-Catabolic Kinetics Module (Lexogen, 062.24) was used for SLAM-seq. Cells were transduced with lentiCRISPR_v2 expressing negative control guides (sgNT1 and sgNT2) or guides targeting *CNOT3* (sg*CNOT3*-1 and sg*CNOT3*-2), selected in puromycin for four days, and seeded into 6-well plates coated with poly-D-lysine. 24 hours later, cells were incubated with 75 μM 4-SU for 24 hours, during which media was exchanged every 3 hours, to label newly synthesized RNA. Cells were then washed 2 times with PBS, and fresh medium containing 10 mM UTP was added to stop labelling. Cells were harvested at 0, 1, 2, 4, 8, 12 hours after UTP addition. Total RNA was isolated and treated with IAA according to the SLAMseq Kinetics Kit manufacturer’s instructions. SLAM-seq libraries were constructed using the QuantSeq 3’ mRNA-Seq V2 Library Prep Kit FWD with UDI 12 nt Set B1 (Lexogen, 192.24). Reads were aligned to human genome assembly GRCh38 and analyzed as described previously (31, 64).

### Measurement of reporter mRNA stability

The 42×R_CGG/CGA/AGG_ and 42×control gene fragments were synthesized by IDT (sequences provided in Table S2). The EGFP sequence in piggyBac transposon vector tetOFF-*EGFP*^PTC35^ (63) between the KpnI and PmlI sites was excised, and replaced with the 42×R_CGG/CGA/AGG_ or 42×control fragments. The resulting plasmids, together with pCMV-hyPBase plasmid (65) which encodes the piggyBac transposase, were co-transfected into HEK293T cells, and cells were selected in medium with 5 μg/mL blasticidin (ThermoFisher Scientific) for 21 days to generate stable cell lines. To measure mRNA stability, cells were treated with 1 μg/mL doxycycline (Sigma) for 0, 2, 4, or 6 hours. qRT-PCR was performed to assess reporter mRNA abundance relative to *GAPDH* mRNA at each time point.

### qRT-PCR

Total RNA was isolated with the RNeasy Mini kit (QIAGEN). cDNA was synthesized from 1 μg total RNA using PrimeScript RT Master Mix (Clontech). qRT-PCR was performed with the Power SYBR^™^ Green Master Mix (ThermoFisher Scientific). mRNA expression was normalized to *GAPDH* mRNA. The sequences of some qRT-PCR primers are from PrimerBank (66). Primer sequences are provided in Table S2.

### Western blotting

Cells were lysed in RIPA buffer (150 mM NaCl, 1% NP-40, 0.5% sodium deoxycholate, 0.1% SDS, 50 mM Tris-HCl pH 7.4, and 1×EDTA-free protease inhibitor cocktail). Lysate was cleared by centrifugation and supplemented with 1×NuPAGE LDS Sample Buffer. For analysis of proteins in sucrose gradient fractions from cell lysates, proteins were precipitated using trichloroacetic acid (TCA) and resuspended in 1×NuPAGE LDS Sample Buffer (Invitrogen). For analysis of proteins in sucrose gradient fractions from *in vitro* translation reactions, proteins were precipitated using the High Efficiency Protein Precipitation Kit (Invent, WA-006). Proteins were separated by SDS-PAGE electrophoresis, transferred to nitrocellulose membranes (0.25 μm, ThermoFisher Scientific), and detected using an infrared fluorescent antibody detection system (LI-COR). Antibodies used for western blotting: CNOT3 antibody (ProteinTech, 11135–1-AP; RRID: AB_2229682), CNOT1 antibody (Cell Signaling, #44613; RRID: AB_2783868), CNOT2 antibody (Cell Signaling, #34214; RRID: AB_2799049), CNOT4 antibody (Proteintech, 12564–1-AP; RRID:AB_2082457), M2 Flag antibody (Sigma, F3165; RRID: AB_259529), RPS25 antibody (Novus Biologicals, NBP1–80802; RRID: AB_11012564), RPL5 antibody (Abcam, ab157099), and RPL11 antibody (ProteinTech, 16277–1-AP; RRID: AB_2181292).

### Measurement of mitochondrial translation

1×10^6^ Jurkat cells were washed 3 times with methionine-free medium, followed by incubation in methionine‐free medium containing 10% FBS and 100 μg/mL anisomycin for 30 minutes to block cytosolic translation (67, 68). 500 μM HPG (ThermoFisher Scientific, C10186) was then added, followed by an additional 30 minute incubation. Media was then replaced with ice-cold buffer A containing 10 mM HEPES, 10 mM NaCl, 5 mM KCl, 10% sucrose, and 0.005% digitonin, for 2 minutes on ice, followed by 15 seconds in buffer A without digitonin. Cells were fixed by adding 4% PFA in PBS for 30 minutes at room temperature. After fixation, cells were washed with PBS for 5 minutes, then quenched with 150 mM glycine in PBS for 15 minutes. Cells were blocked and permeabilized in staining solution (5% BSA and 0.1% Triton X‐100 in PBS) (three solution changes, 5 minutes for each). After a brief wash with 3% BSA in PBS, cells were click labeled with 5 μM ATTO 488‐azide (sigma) for 20 minutes using Click‐iT Cell Reaction Buffer Kit (ThermoFisher Scientific). After a quick wash with Intercept (PBS) blocking buffer (LI-COR), cells were incubated with TOMM20 antibody [Santa Cruz, sc-17764; RRID: AB_628381; 1:200 dilution in Intercept (PBS) blocking buffer] at 4° C for 1 hour, and then anti-mouse AF647 (ThermoFisher Scientific, A-21235; RRID: AB_2535804, 1:1000 dilution in Intercept (PBS) blocking buffer] at 4° C for 30 minutes. Cells were then stained with DAPI diluted in Intercept (PBS) blocking buffer for 5 minutes at room temperature. After three washes with 3% BSA in PBS, cells were resuspended in 20 μl mounting medium (SlowFade^™^ Diamond Antifade Mountant with DAPI, ThermoFisher Scientific, S36968), and transferred to a slide, and imaged on a Zeiss LSM980 confocal microscope.

### Measurement of mitochondrial content by MitoTracker

1×10^6^ cells were collected in a microcentrifuge tube and washed with PBS twice. Cells were resuspended in 1 mL PBS, stained with MitoTracker (ThermoFisher Scientific, M7574) according to the manufacturer’s instructions, and analyzed using and Accuri C6 Flow Cytometer (BD Biosciences).

### Preparation of mRNAs for *in vitro* translation

#### Generation of DNA template for in vitro transcription

(1) The plasmid XLone-GFP (Addgene #96930) (69) was modified such that it carried a cassette consisting of a T7 promoter and Kozak sequence, followed by DraIII and BlpI sites, creating plasmid XLone-T7. A similar plasmid with a T7 promoter, Kozak sequence, and HA-tag, followed by DraIII and BlpI sites, termed XLone-T7-HA, was also generated. Sequences of T7 and T7-HA cassettes are provided in Table S2.

(2) 41×LR_CGG_D, 41×LR_CGA_D, 41×LR_CGU_D, 41×LK_AAA_D, 41×LK_AAG_D, and 41×LM_AUG_D DNA templates were generated using a method we developed, called “repeat PCR-ligation extension”. Briefly, oligos containing a BsrD1 site, followed by six copies of each repeat, followed by a BseR1 site, were synthesized. Each oligo was cloned into the DraIII and BlpI sites in XLone-T7 using the NEBuilder^®^ HiFi DNA Assembly Master Mix (New England Biolabs, E2621L). The sequence from the T7 promoter to 31 bp downstream of the Blp1 site was amplified by PCR with Q5 High-Fidelity DNA Polymerase (New England Biolabs, M0491L) using primers T7-F1 and T7-R1 (all oligonucleotide sequences provided in Table S2). Half of the PCR product was digested with BsrD1 followed by purification of the 3’ fragment, and the other half of the PCR product was digested with BseR1 followed by purification of the 5’ fragment. The purified 3’ and 5’ fragments were ligated using the Quick Ligation^™^ Kit (New England Biolabs, M2200L). The ligation product was purified using the QIAquick PCR Purification Kit (Qiagen, 28106) and size-selected on an agarose gel. The resulting 11×Repeats was PCR amplified, using primers T7-F1 and T7-R1, and used for another round of repeat extension following the same strategy. Three rounds of extension produced 41×Repeats. A poly(A) tail was added to the DNA template by PCR using primers T7-F1 and poly T-R1. The final PCR product was purified using the QIAquick Gel Extraction Kit (Qiagen, 28706), followed by further purification using a DNA Clean&Concentrator-5 kit (Zymo Research, D4003). The sequence of the final PCR product was confirmed by Sanger sequencing. As the amplification error rate of Q5 High-Fidelity DNA Polymerase is 5×10^−7^, the percentage of correct DNA copies is estimated to be greater than 99%.

(3) 73×R-CGG/CGA/AGG and 73×control DNA templates were generated using the same “repeat PCR-ligation extension” strategy, with two modifications: oligos containing nineteen CGG/CGA/AGG or nineteen I/K/N/F/M/Y codons were synthesized (sequences provided in Table S2), and the oligos were cloned into the BspE1 and BlpI sites of Xlone-T7-HA. Two rounds of extension were performed to generate 73×Repeats.

#### In vitro transcription and capping of mRNA

mRNA was generated by *in vitro* transcription using the HiScribe^®^ T7 High Yield RNA Synthesis Kit (New England Biolabs, E2040S). mRNA was purified using the Monarch^®^ RNA Cleanup Kit (New England Biolabs, T2050S), capped with Faustovirus Capping Enzyme (New England Biolabs, M2081L) at 42° C for 1 hour, and purified using the Monarch^®^ RNA Cleanup Kit. mRNA was stored in −80° C.

### Generation of tRNAs by *in vitro* transcription

Each tRNA used for *in vitro* translation or mutagenesis represented the most abundant isodecoder expressed in HEK293T cells for a given codon. DNA oligos containing mature tRNA sequence (with CCA at the 3’ end) were synthesized (sequences provided in Table S2) and used as templates for PCR with a forward primer, containing a T7 promoter, annealing to the 5’ end and a reverse primer annealing to the 3’ end of the tRNA. The first two nucleotides of the reverse primer were 2′-O-methylated to improve the homogeneity of the tRNA 3’ end, as reported previously (70). PCR products were purified using the QIAquick Gel Extraction Kit (Qiagen, 28706) followed by further purification using the DNA Clean&Concentrator-5 kit (Zymo Research, D4003). *In vitro* transcription was performed using the HiScribe^®^ T7 High Yield RNA Synthesis Kit and RNA was purified using the Monarch^®^ RNA Cleanup Kit (New England Biolabs, T2050S). Typically, 80–150 μg tRNA was produced from one 20 μL reaction. tRNA was aliquoted and stored at −80° C.

### Expression and purification of RARS-6×His and HA-MARS

Rosetta^™^ 2(DE3) pLysS Competent Cells were transformed with plasmid RARS_pNIC-Bio3 (Addgene #153055) and plated onto LB plate containing kanamycin (100 μg/mL) and chloramphenicol (33 μg/mL). Clones were picked and grown in 5 mL LB medium at 37° C overnight. 2 mL culture was inoculated into 1 liter LB medium and shaken at 37° C for 1–2 hours. When the OD600 of the culture reached 0.06 (measured by Nanodrop), culture was placed on ice for 30 minutes, followed by overnight shaking at 37° C in the presence of 0.2 mM IPGT. Cells were harvested by centrifugation at 3,000 g for 5 minutes, washed with ice-cold PBS, and then resuspended and sonicated in 20 mL lysis buffer [0.5 M NaCl, 1 mM MgCl_2_, 50 mM Tris-HCl (pH 7.5), 10 mM β-mercaptoethanol, 5 mM imidazole, and 1mM PMSF]. Lysate was cleared by centrifugation at 22,000 g for 30 minutes. The resulting supernatant was sequentially filtered through 5 μm and 0.2 μm filters. RARS-6×His protein was captured by incubating the precleared lysate with 1 mL Ni-NTA resin (ThermoFisher Scientific, 88221) at 4° C with rotation for 1 hour. Resin was collected by centrifugation, resuspended in 20 mL wash buffer [1 M NaCl, 1 mM MgCl_2_, 50 mM Tris-HCl (pH 7.5), 10 mM β-mercaptoethanol, and 25 mM imidazole], and transferred to a gravity column. 10 mL elution buffer [0.5 M NaCl, 1 mM MgCl_2_, 50 mM Tris (pH 7.5), 10 mM β-mercaptoethanol, and 0.5 M imidazole] was added to the gravity column to elute RARS-6×His protein. Protein was concentrated with a protein concentrator (30K MWCO, ThermoFisher Scientific, 88529) and stored at −80° C.

HA-MARS was expressed and purified from HEK293T cells. HEK293T cells growing in a 15-cm dish were transfected with 10 μg pcDNA3-HA-MARS plasmid (Addgene #10716) using FuGENE HD (Promega). Two days after transfection, cells were harvested by scraping and washed with ice-cold PBS. Cells were resuspended in PBS supplemented with 1×EDTA-free protease inhibitor cocktail (Roche) and 1% NP-40, and sonicated. Lysate was cleared by centrifugation at 22,000 g for 30 minutes. The resulting supernatant was sequentially filtered through 5 μm and 0.2 μm filters. Cleared lysate was incubated with anti-HA Magnetic Beads (ThermoFisher Scientific, 88836; RRID: AB_2749815) at room temperature with rotation for 1 hour. Beads were washed with PBST. HA-MARS protein was eluted from beads using 2 μg/μL HA peptide in PBS. HA-MARS protein was concentrated with an Amicon Ultra Centrifugal Filter (30K MWCO, Millipore, UFC503008) and stored at −80° C.

### tRNA aminoacylation assay

tRNA aminoacylation efficiency was measured by biotinylation-streptavidin conjugation to the α-amine of the aminoacyl group followed by gel electrophoresis to separate the aminoacyl-tRNA and tRNA as described (71). Briefly, *in vitro* transcribed tRNAs were refolded by incubating at 95° C for 2 minutes, 22° C for 3 minutes, and 37° C for 5 minutes. 30 μL reactions [3 μg refolded tRNA, 3 μg recombinant RARS-6×His or HA-MARS, 3.3 mM ATP, 100 μM (L)-Arginine or (L)-Methionine, 50 mM HEPES (pH 7.3), 25 mM KCl, 15 mM MgCl_2_, 0.1 mM DTT, 0.75 μL RNasin (Promega)] were incubated at 37° C for 1 hour. Total tRNA was purified with the RNA Clean & Concentrator-5 kit (Zymo Research, R1016) and eluted in 30 μL ddH_2_O. To biotinylate the α-amine of aminoacyl-tRNA with sulfo-NHS-biotin (ThermoFisher Scientific, 21217), the 30 μL aminoacyl-tRNA was combined with 30 μL 120 mM HEPES (pH 8.0) containing 600 μg sulfo-NHS-biotin, followed by incubation at 4° C for 1 hour. Total tRNA was again purified with the RNA Clean & Concentrator-5 kit and eluted in 30 μL ddH_2_O. 1 μg of the purified reacted tRNA was combined with 20 μL streptavidin (1 μg/μL, New England Biolabs, N7021S), and incubated at room temperature for 20 minutes, followed by separation on a 2% agarose gel. Ethidium bromide staining was used to visualize charged and uncharged tRNA on the gel.

### Preparation of translation-competent cell lysate

Twenty 15 cm plates of 90–95% confluent HEK293T cells were treated with 200 nM ISRIB (Sigma, SML0843) for 90 minutes. Cells then were dissociated from dishes by scraping in media and centrifuged at 800 g at 4° C for 3 minutes, followed by four washes in ice-cold PBS containing 200 nM ISRIB. Thereafter, all handling was performed in a 4° C cold room. Cells were resuspended in an equal volume of ice-cold hypotonic lysis buffer with 10 mM HEPES (pH 7.3), 10 mM KAc, 0.5 mM MgAc_2_, 1 mM DTT, 2 mM D-glucose, 200 nM ISRIB, and 1×protease inhibitor cocktail (e.g. 1 mL lysis buffer for a 1 mL cell pellet). Cells were placed on ice for 10 minutes and homogenized with ten strokes of a Wheaton homogenizer. The lysis process was monitored by trypan blue staining until 90–95% of the cells were lysed (excessive homogenization compromises the translational activity of the lysate). Lysate was centrifuged at 16,000 g at 4° C for 10 minutes. The supernatant was transferred to a new tube and incubated with 1 mM CaCl_2_ and 0.8 u/μL Micrococcal Nuclease (ThermoFisher Scientific, 88216) at 20° C for 10 minutes. Micrococcal Nuclease was quenched by addition of EGTA to a final concentration of 10 mM and mixing by gentle inversion. Nuclease-treated lysate was centrifuged at 16,000 g at 4° C for 5 minutes to remove precipitates. The lysate was aliquoted, snap frozen, and stored at −80° C.

### Generation of HEK293T cell lysate expressing WT or mutant CNOT3

The *BSD* gene in plasmid pLenti-CMV-Blast (w263–1) (Addgene #17486) (72) was replaced by the *PuroR* gene to generate pLenti-CMV-Puro. WT or mutant CNOT3–3×Flag was then cloned into pLenti-CMV-Puro using the Esp3I and BamH1 sites. Lentivirus was generated using HEK293T cells as described previously (63). HEK293T cells were transduced with lentivirus in the presence of 8 μg/mL of polybrene (Sigma, S2667). Two-days post-transduction, cells were selected with 0.5 μg/mL puromycin for 6 days. Cells then were collected, and translation-competent lysate was prepared as described above.

### *In vitro* translation

*In vitro* translation conditions were optimized using luciferase mRNA to determine the optimal concentration of K^+^, Mg2^+^, Spermidine, and mRNA, as well as the optimal incubation time and temperature. Translation reactions were most efficient when assembled as follows: 40% HEK293T lysate, 10% CNOT3–3×Flag expressing HEK293T lysate, 15 mM HEPES (pH 7.3), 0.2 mM MgCl_2_, 70 mM KCl, 28 mM KAc, 6 mM Creatine phosphate (Sigma, 10621714001), 102 ng/μL Creatine Kinase (Sigma, 10127566001), 0.4 mM amino acids mixture (Promega, L4461), 1 U/μL RNasin (Promega), 0.2 mM spermidine (Sigma, S2626), and 80 ng/μL mRNA. Reaction was incubated at 30° C for 40 minutes.

For *in vitro* translation reactions supplemented with *in vitro* transcribed tRNA, the creatine phosphate concentration was increased to 18 mM. Before adding to the *in vitro* translation reactions, *in vitro* transcribed tRNAs were refolded by incubating at 95° C for 2 minutes, 22° C for 3 minutes, and 37° C for 5 minutes. 10 μg tRNA^Met^ or mutants derived thereof were added to 100 μL reactions. For other tRNAs, 2 μg tRNA was added to 100 μL reactions, unless otherwise specified.

### Purification of CNOT3-bound ribosomes translating 41×LR_CGG_D mRNA

2 mL *in vitro* translation reaction, containing 80 ng/μL 41×LR_CGG_D mRNA, 10% CNOT3–3×Flag expressing HEK293T lysate, and 40% WT HEK293T lysate, was used for the purification of CNOT3-bound ribosomes for structural studies. Reactions were separated on a 5–50% sucrose gradient containing 20 mM HEPES (pH 7.5), 100 mM KCl, 5 mM MgCl_2_, and 1×EDTA-free protease inhibitor cocktail. Polysome fractions were collected and combined. To remove sucrose, 3 mL 1× gradient buffer [20 mM HEPES (pH 7.5), 100 mM KCl, 5 mM MgCl_2_, 1×EDTA-free protease inhibitor cocktail] was added to the polysome sample. A protein concentrator (30K MWCO, ThermoFisher Scientific, 88529) was then used to reduce sample volume to 2 mL. 2 mL 1× gradient buffer was then added to the sample, and further concentration to 1.5 mL was performed. 100 μL anti-DYKDDDDK Magnetic beads (ThermoFisher Scientific, A36797) were added to the concentrated polysome sample, followed by incubation at 4° C for 1.5 hours with slow rotation. Beads were washed with ice-cold wash buffer (1× gradient buffer containing 0.01% NP-40) four times. CNOT3–3×Flag-Ribosome complex was eluted from beads with 200 μL elution buffer (1× gradient buffer containing 0.01% NP-40, 1 mM DTT, and 1.5 mg/mL 3×Flag peptide) at 16 °C for 30 minutes with 300 RPM shaking. The 200 μL eluate was transferred to a new microcentrifuge tube and combined with 200 μL 1× gradient buffer supplemented with 0.01% NP-40 and 1 mM DTT. Sample was concentrated to 100 μL using a protein concentrator (Millipore, 30KD, 0.5 mL). Then 200 μL 1× gradient buffer supplemented with 0.01% NP-40 and 1 mM DTT was added to sample, and sample was concentrated to 50 μL. The concentration of the purified ribosome complex was estimated to be ~174 nM, as determined by absorbance at 260 nm measured by Nanodrop. Before grid preparation, the sample was diluted to 87 nM using Ribo-buffer B [50 mM Bis-Tris-Propane HCl pH 8.0, 125 mM NaCl, 25 mM KCl, 10 mM MgCl2, 1 mM TCEP, and 0.01% (w/v) NP-40].

### Northern Blotting

Total RNA was isolated from proteinase K-treated *in vitro* translation reactions programmed with 41×LR_CGG_D mRNA (lysate), combined polysome fractions (input), or CNOT3-bound polysomes (enriched by anti-Flag IP). RNA was separated on 8% TBE-Urea polyacrylamide gels and transferred to BrightStar-Plus nylon membranes (Invitrogen). Membranes were UV-crosslinked (254 nm UV crosslinker at 120 mJ/cm2) and prehybridized with ULTRAhyb-Oligo hybridization buffer (Invitrogen). Blots were hybridized overnight with biotin-labeled oligonucleotide probes (Sigma). Membrane was washed, incubated with Streptavidin-IR800 (LI-COR), and imaged on a LI-COR Odyssey. Probe sequences are provided in Table S2.

### Cryo-EM grid preparation and Data Collection

Cryo-EM grids were prepared by applying 3.5 μL of the CNOT3–80S ribosome complex sample to glow-discharged (Pelco easyGlow – 30s at 30mA) continuous carbon (2.2–2.5 nm) coated Quantifoil R2/1 300-mesh grids. Grids were blotted and frozen in liquid ethane using a Mark IV Vitrobot (FEI) set at 4 °C and 100% humidity. Micrographs were acquired on a Titan Krios (FEI) operated at 300 kV using a Falcon 4i direct electron detector equipped with a cold-field emission gun (cold-FEG) and a slit width of 10 eV on a GIF-Quantum energy filter with fringe free illumination. Automated data collection, totaling 11907 movies was performed using SerialEM (73) with a defocus range of −0.6 to −1.9 μm and a pixel size of 0.936 Å. Micrographs were dose fractionated into 496 frames each under a dose rate of 8.39 e-/pixel/s with a total exposure time of 2 seconds and a total dose of approximately 16.78 e-/pixel.

### Cryo-EM data processing

Data were processed using Relion 4.0 (74). During motion correction using RELION’s own implementation we collapsed 496 frames into 20 frames and CTF estimation was performed using CTFIND-4.1 (75). About 300 particles were picked to generate initial 2D classes to serve as templates for automated particle picking from the 11154 micrographs selected after CTF correction. 810K particles were picked and extracted, binned 4 times, and used for 2D classification. Of these, 645K particles were selected for 3D classification. Classes representing intact 80S particles were combined and re-extracted at the original pixel size of 0.936 Å, resulting in a total of 378K particles. CTF and 3D refinement was performed with an imposed C1 symmetry and resulted in a reconstruction with a resolution of 2.54 Å without postprocessing. Local skip-align 3D classification (T-factor of 65) was performed using a mask surrounding the CNOT3/tRNA region. From this, 276K particles with the best local resolution were selected for final refinement and post-processing to an overall resolution of 2.00 Å. The final resolution was estimated by applying a soft mask and was calculated using the gold-standard Fourier shell correlation (FSC) = 0.143 (76). Local resolution was generated using the ResMap (77) wrapper within Relion 4.0.

### Cryo-EM model building and refinement

PDBs 6QZP (78), 8GLP and 8G5Y (38) were used to generate a starting model for the 80S, tRNA^Arg, CCG−1^ was built de-novo using the tRNA from PDB 8ISS (45) as a guide. The CNOT3 Alphafold model was rigid-body fitted into the final map density (79). The positions of rRNA (80) and tRNA (80) modifications were individually checked in the initial models and corrected if necessary. After manual rebuilding in COOT (81), the final model was refined in PHENIX using phenix.real_space_refine (82) with Ramachandran and secondary structure restraints. Restraints for the connection between the P-site tRNA and the nascent chain were generated using Acedrg (83). Restraint files for the isoaspartate residue in uS17 were kindly provided by Amos Nissley and Jamie Cate (UC Berkeley). The CNOT3/tRNA^Leu,UAA^ complex from PDB 8BHF (19) was rebuilt in COOT (81) using the HsCNOT3 model and tRNAs from PDBs 8JOZ (84) and 7O80 (85) as guides. The original tRNA sequence from 8BHF (ACCAGGAUGGCCUAGUGGUUAAGGCGUUGGACUUAAGAUCCAAUGGACAUGUGUCCGCGUCGGUUUUCGAACCCCA) was replaced with the sequence for rabbit tRNA^Leu,UAA−1−1^ (ACCAGGAUGGCCGAGUGGUUAAGGCGUUGGACUUAAGAUCCAAUGGACAUGUGUCCGCGUGGGUUCGAACCCCACUCCUGGUA). The final model lacks D-loop nucleotides 19, 20, and the variable loop nucleotides 46–57, and fixes the register shift in the original model. The updated model was refined in PHENIX against map EMD-16052 using phenix.real_space_refine (82) using Ramachandran and secondary structure restraints. Figures were generated with UCSF Chimera X (86) and the PyMOL Molecular Graphics System (Version 2.0 Schödinger, LLC.).

## Supplementary Material

Supplementary Material

Supplementary Table S2

## Data Availability

All high-throughput sequencing data has been deposited in GEO (Accession GSE268325). The cryo-EM density maps and models have been deposited in EMDB and PDB databases under accession codes: EMD-45170/PDB-9C3H (human 80S/CNOT3/tRNA^Arg,CCG^ complex) and PDB-93CI for the rebuilt CNOT3/tRNA^Leu,UAA^ structure. All reagents generated in this study are available upon request from J.T.M with a completed Materials Transfer Agreement.
