# Supplementary material for: Specific tRNAs promote mRNA decay by recruiting the CCR4-NOT complex to translating ribosomes
Source: Science. Author manuscript; Available in PMC 2024 Nov 22. (PMC11583848; doi:10.1126/science.adq8587)
Supplement: Supplementary Material [file NIHMS2027590-supplement-Supplementary_Material.pdf]

Science
AAAS

Supplementary Materials for

Specific tRNAs promote mRNA decay by recruiting the CCR4-NOT complex to translating ribosomes

Xiaoqiang Zhu, Victor Emmanuel Cruz, He Zhang, Jan P. Erzberger, and Joshua T. Mendell

Corresponding authors: Joshua.Mendell@UTSouthwestern.edu; Jan.Erzberger@UTSouthwestern.edu

The PDF file includes:

Figs. S1 to S12
Table S1

Other Supplementary Materials for this manuscript include the following:

Table S2 (Excel)

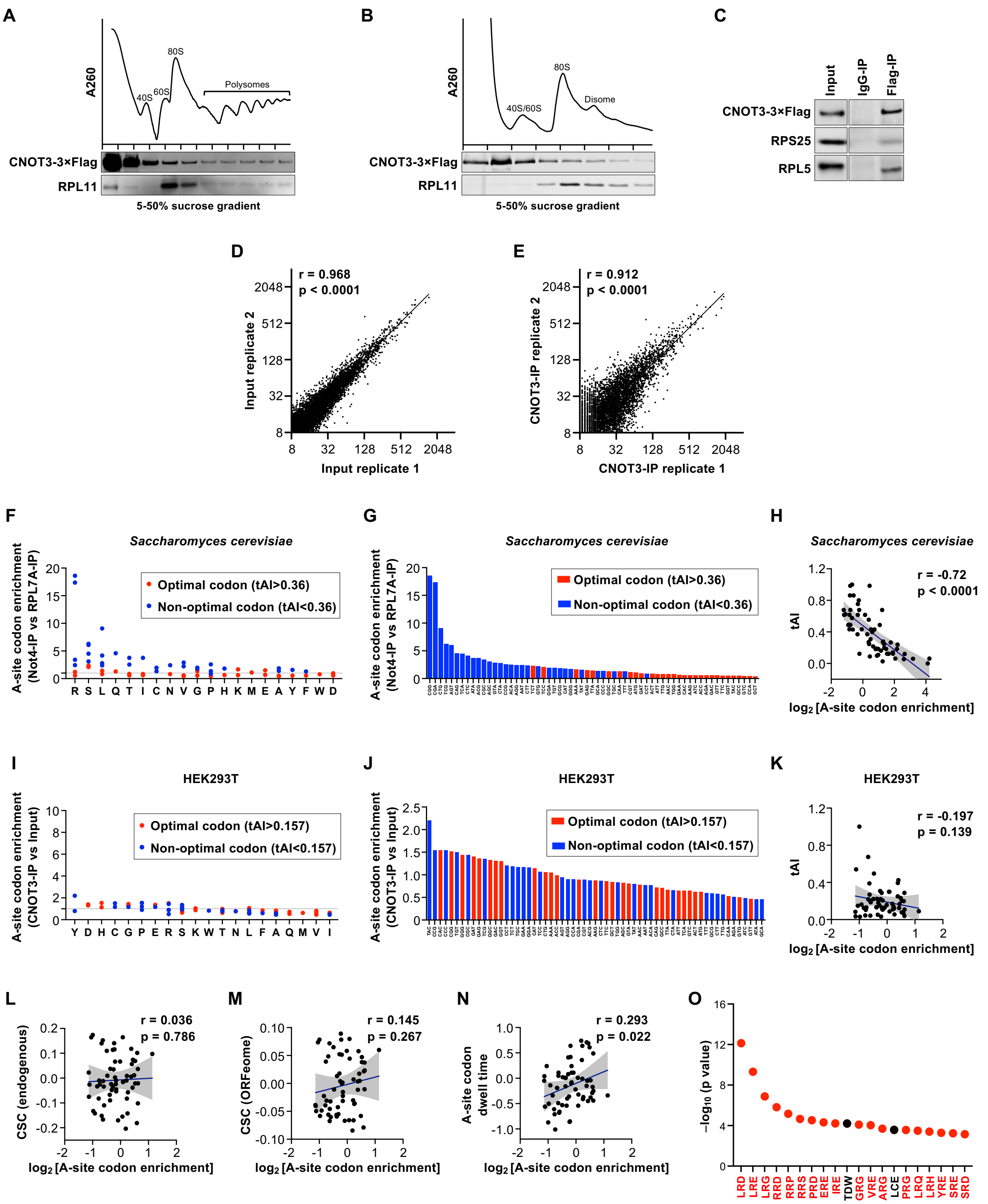

Fig. S1. Analysis of ribosomal recruitment of Not5/CNOT3, related to Fig. 1. (**A** and **B**) Sucrose density gradient profiles of lysates from HEK293T cells expressing CNOT3-3×Flag and western blot analysis of fractions without (A) or with RNase A treatment (B). (**C**) Western blot analysis of CNOT3-3×Flag or control immunoprecipitates. Representative results from n=3

biological replicates shown for sucrose density gradient profiles and Flag IP. (**D** and **E**) Reproducibility of selective ribosome profiling data across biological replicates. (**F** and **G**) Codon enrichment in the ribosomal A-site of Not4-bound ribosomes in *S. cerevisiae* (*14*), grouped by amino acid (F) or codon (G). (**H**) Pearson correlation of codon enrichment in the ribosomal A-site of Not4-bound ribosomes and tAI (*29*) in *S. cerevisiae*. (**I** and **J**) Codon enrichment in the ribosomal A-site of CNOT3-bound ribosomes in HEK293T cells, grouped by amino acid (I) or codon (J). (**K**) Pearson correlation of codon enrichment in the ribosomal A-site of CNOT3-bound ribosomes and tAI (*20*) in HEK293T cells. **(L** and **M)** Pearson correlation between A-site codon enrichment in CNOT3-bound ribosomes and codon stability coefficient (CSC) in HEK293T cells. The CSC was calculated previously using either the half-lives of endogenous mRNAs or an ORFome library (*21*). (**N**) Pearson correlation of codon enrichment in the ribosomal A-site of CNOT3-bound ribosomes and A-site dwell time (*25*) in HEK293T cells. (**O**) Top 20 enriched tripeptides encoded by CNOT3-bound ribosomal footprints.

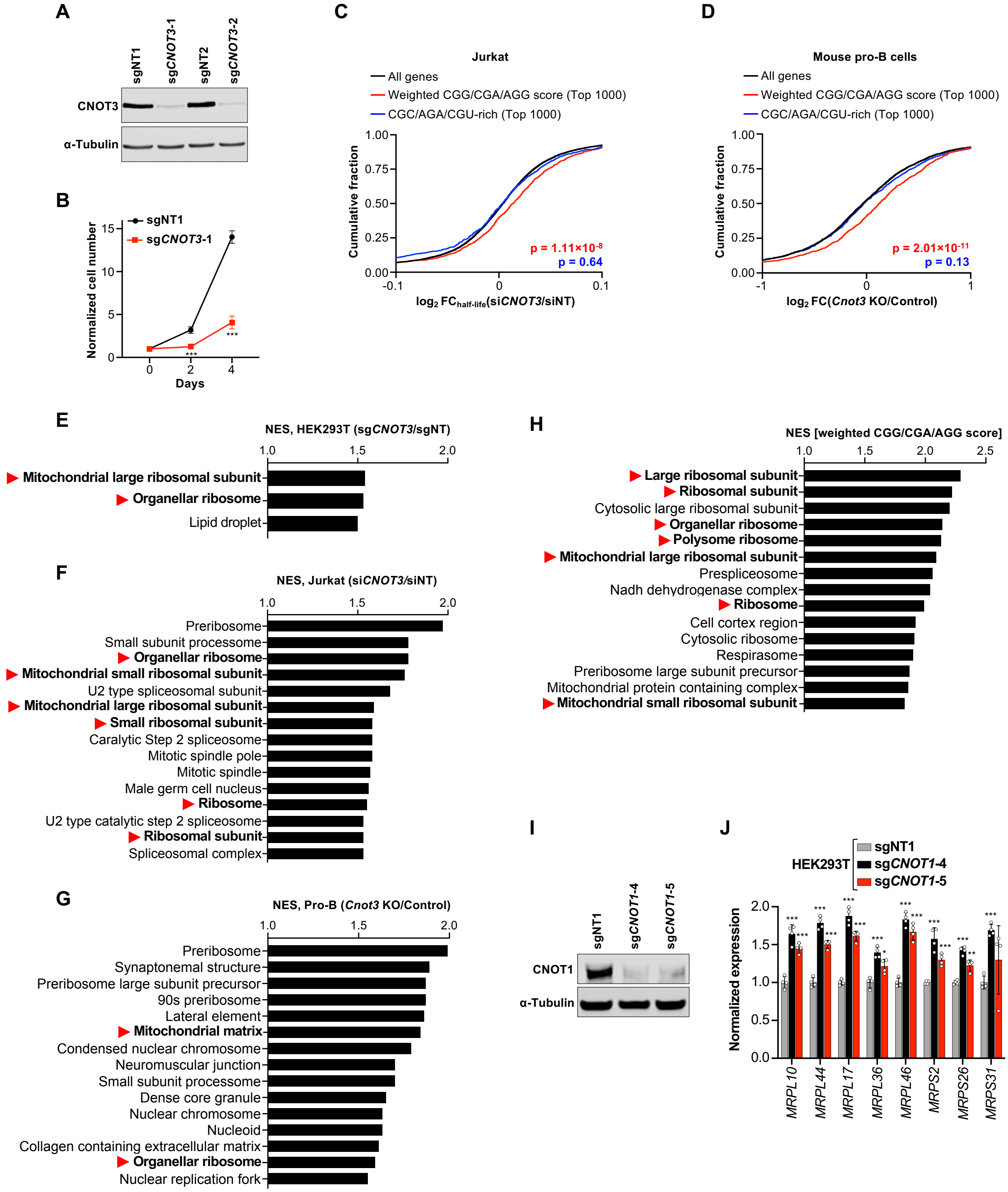

Fig. S2. Regulation of CGG/CGA/AGG-rich transcripts by CNOT3, related to Fig. 2. (A) Western blot analysis of CNOT3 protein in HEK293T cells after lentiviral delivery of Cas9 and non-targeting (NT) or *CNOT3*-targeting sgRNAs. **(B)** Growth of HEK293T cells. Cells were transduced with lentivirus expressing Cas9 and non-target (NT) or *CNOT3*-targeting sgRNA. Two days post-transduction, cells were selected with 0.5 μg/mL puromycin for 4 days. Day 0

represents the time point at which puromycin was removed and proliferation measurements were initiated. n=3 biological replicates (mean ± SD shown). *P* values were calculated by student's t test. ****P*<0.001. (**C**) CDF plot showing the fold-change in half-lives of mRNAs with high weighted CGG/CGA/AGG scores or mRNAs rich in CGC/AGA/CGU arginine codons in CNOT3-depleted Jurkat cells. mRNA stability measurements in Jurkat cells after CNOT3 knockdown were reported previously (*33*). mRNAs rich in CGC/AGA/CGU codons that also had a high weighted CGG/CGA/AGG score (top 1000) were excluded from this gene set. *P* values were calculated by one-sided Wilcoxon rank sum test. (**D**) CDF plot showing the fold-change in steady-state abundance of mRNAs in pro-B cells from *Cnot3* knockout mice. mRNA expression in *Cnot3* knockout pro-B cells was reported previously (*34*). (**E** to **G**) GSEA of mRNA decay rate data from *CNOT3*-knockout HEK293T cells (E) or *CNOT3*-knockdown Jurkat cells (F), or steady-state mRNA levels from *Cnot3* knockout pro-B cells (G). All genesets with FDR<0.25 are shown for HEK293T cells, while the top 15 genesets (all with FDR<0.25) are shown for Jurkat and pro-B cells. NES, normalized enrichment score. Red triangles indicate genesets containing mitochondrial ribosomal proteins. (**H**) GSEA showing the top 15 enriched genesets based upon weighted CGG/CGA/AGG score (all with FDR<0.25). **(I)** Western blot analysis of CNOT1 protein in HEK293T cells after lentiviral delivery of Cas9 and non-targeting (NT) or *CNOT1*-targeting sgRNAs. **(J)** qRT-PCR analysis of mitochondrial ribosomal protein mRNAs, normalized to *GAPDH*, in HEK293T infected with lentivirus expressing the indicated sgRNAs. n=4 biological replicates (mean ± SD shown). *P* values were calculated by student's t test. **P*<0.05; ***P*<0.01; ****P*<0.001.

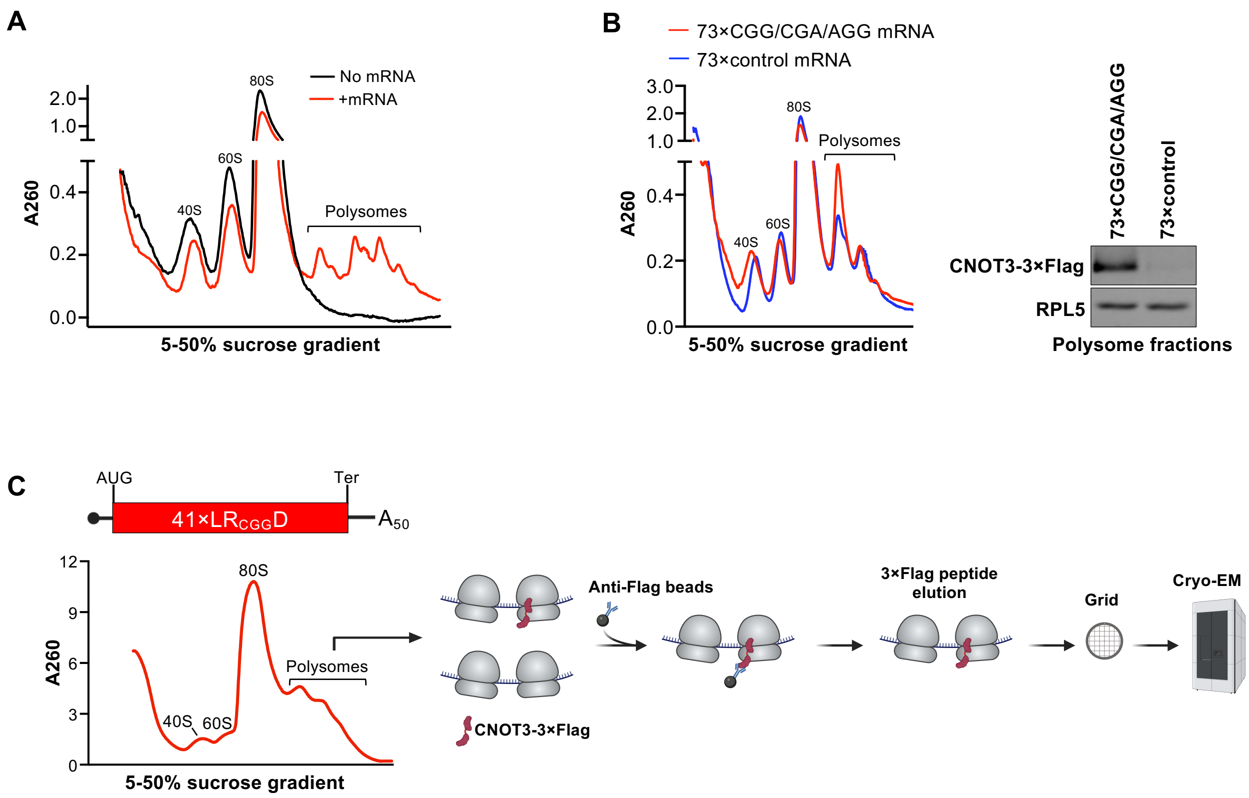

Fig. S3. *In vitro* translation of CGG/CGA/AGG-rich transcripts, related to Fig. 3. (**A**) Sucrose density gradient profile of *in vitro* translation reactions performed with or without added mRNA. (**B**) Sucrose density gradient profiles and western blot analysis of combined polysome fractions from *in vitro* translation reactions assembled on the indicated mRNAs. Representative data from n=3 biological replicates shown. (**C**) Strategy for enrichment of CNOT3-3×Flag-bound ribosomes translating 41×LR$_{CGG}$D mRNA for cryo-EM analysis. Figure created with BioRender.com.

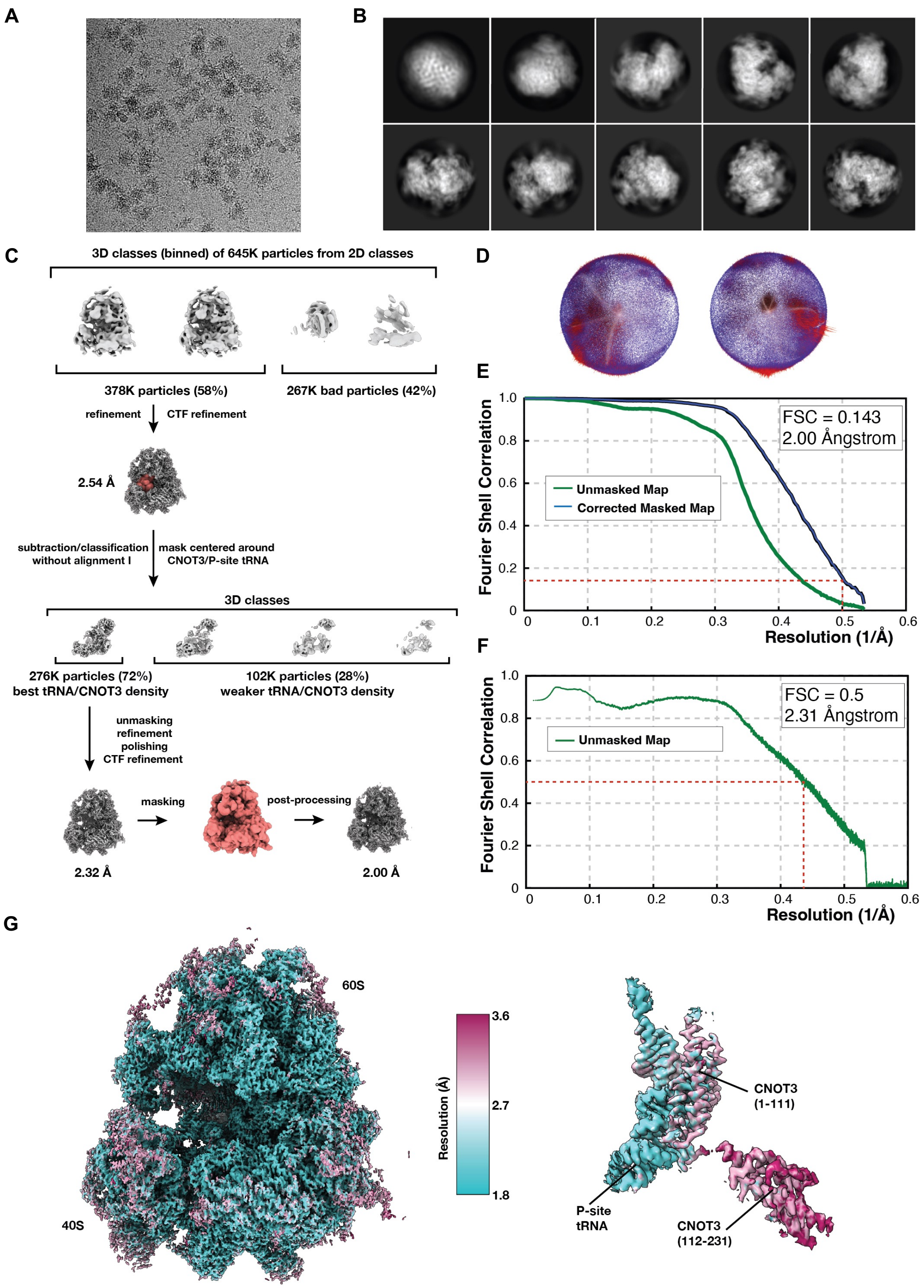

Fig. S4. Cryo-EM analysis of CNOT3-bound ribosomes, related to Fig. 3. (**A**) Representative micrograph used for the 80S ribosome-CNOT3 reconstruction. The clustering pattern suggests

that a subset of polysomes are preserved during sample preparation. (**B**) Representative 2D classes of 80S ribosomes with a broad distribution of orientations. (**C**) Workflow of the 3D classification scheme. Map volumes are shown for all steps and masks (red) indicate the volumes selected for local classification and post-processing. Major sorting and classification criteria, particle numbers and percentages (for each 3D classification step) as well as final map resolutions are indicated. **(D)** Euler angle distribution of particles in the final refinement subset. (**E**) Gold-standard Fourier shell correlation (FSC) curves (masked and unmasked) indicating an overall resolution of 2 Å, approaching the Nyquist limit for our collection setup. (**F**) Model-map Fourier-shell correlation (FSC) curve. (**G**) Overall (left) and CNOT3/tRNA^{Arg,CGG} focused (right) cryo-EM maps colored according to local resolution distribution.

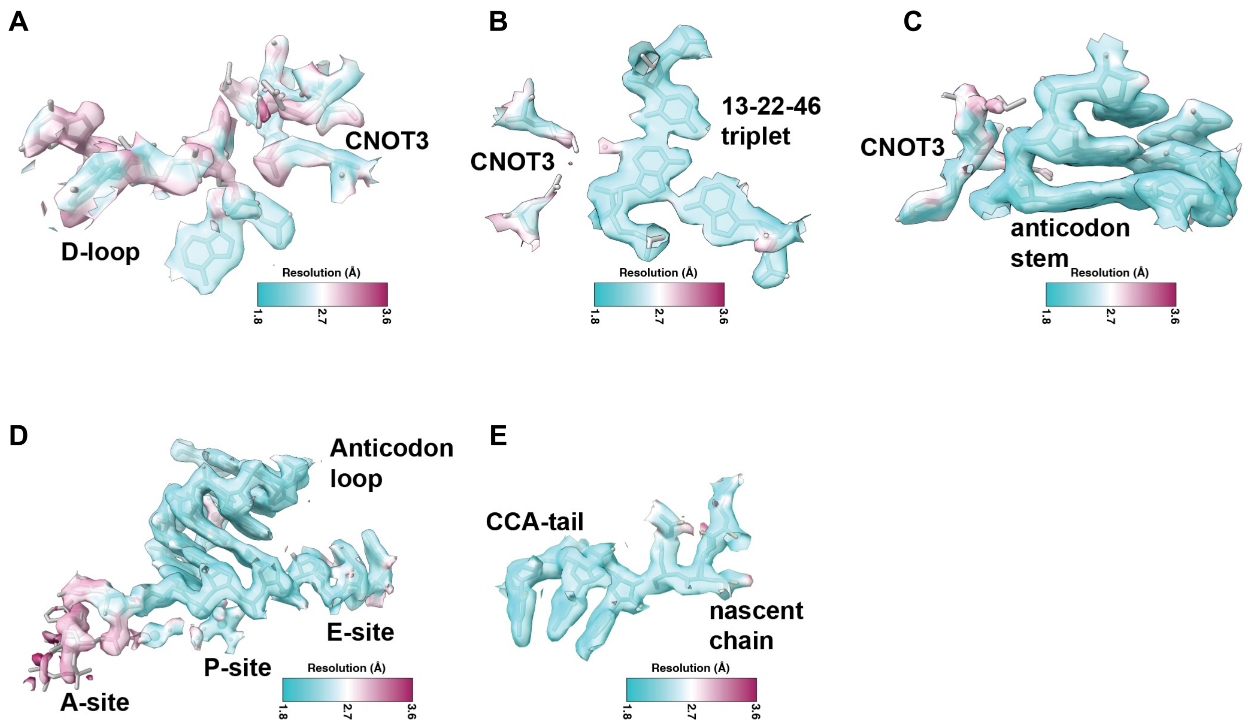

Fig. S5. Local resolution analysis, related to Fig. 3. (A-E) Experimental Cryo-EM density colored according to local resolution focusing on key regions of the complex: (A) CNOT3-D-loop; (B) CNOT3-D-stem; (C) CNOT3-anticodon stem; (D) Codon-anticodon interaction; (E) CCA-tail/nascent chain.

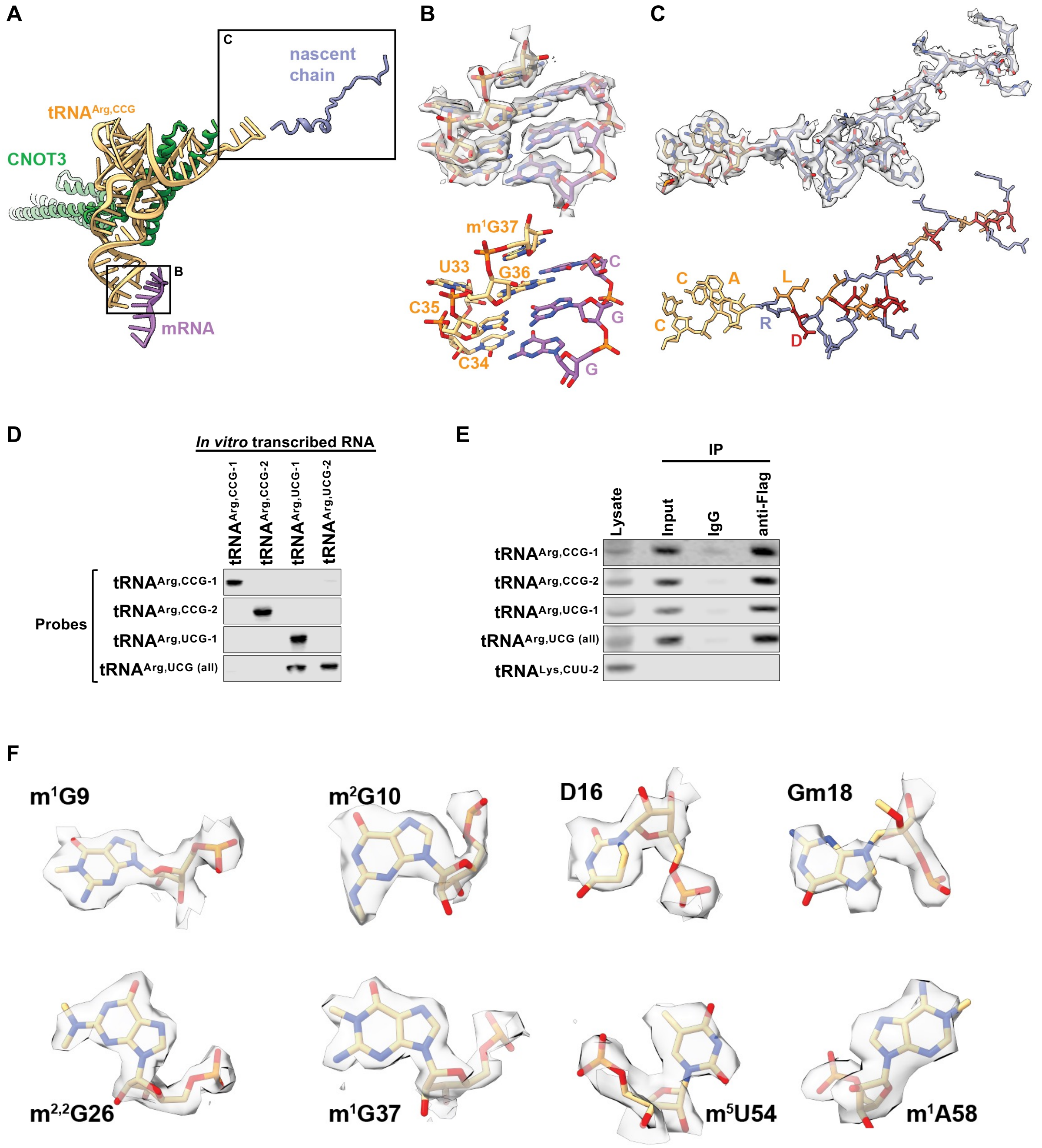

Fig. S6. Analysis of tRNA in the CNOT3-ribosome structure, related to Fig. 3. (**A**) Cartoon model of the CNOT3/tRNA complex highlighting the codon:anticodon base-pairing and the CCA-tail/nascent chain region. (**B** and **C**) Density maps and atomic models of the codon:anticodon base-pairing consistent with $tRNA^{Arg,CCG}$ (B) and nascent chain with the predicted amino acid repeats (C). (**D**) Northern blot analysis of *in vitro* transcribed tRNAs to verify probe specificity. (**E**) Northern blot analysis of arginine tRNAs in CNOT3-bound ribosomes purified from *in vitro* translation reactions programmed with 41×LR_{CGG}D mRNA. Input represents polysome fractions prior to anti-Flag IP. Representative data from n=2 biological replicates shown. (**F**) Molecular structures and cryo-EM densities of select $tRNA^{Arg,CCG-1}$ modifications modeled in our CNOT3-ribosome structure.

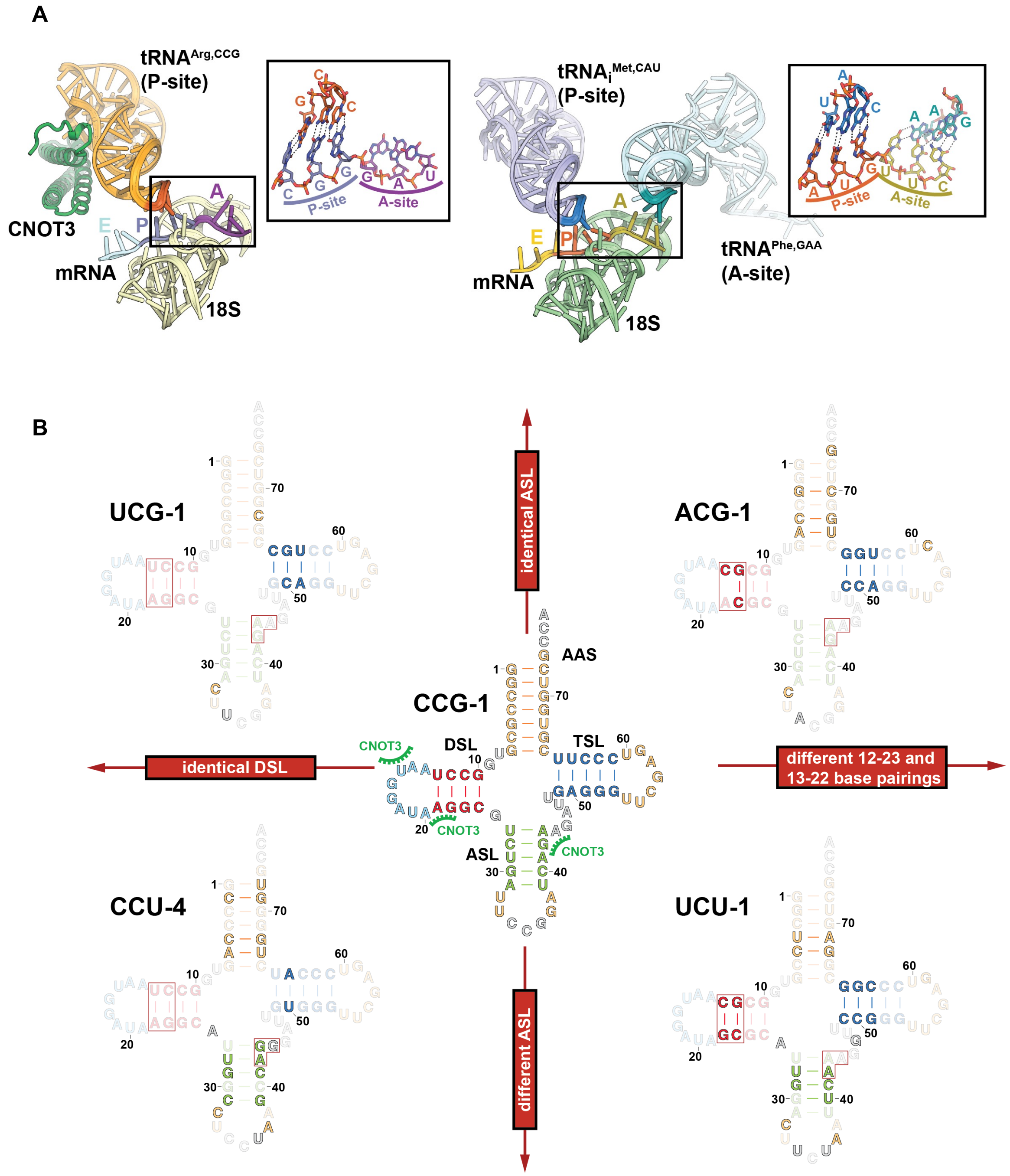

Fig. S7. mRNA configuration in CNOT3-bound ribosomes and arginine tRNA sequences, related to Figs. 3 and 4. (**A**) Cartoon structures and molecular models (insets) showing the configuration of mRNA in our CNOT3-bound ribosome structure (left) compared to the mRNA configuration in ribosomes bound by EEF1A-tRNA^{Phe,GAA}-GTPγS from PDB 8G5Z (GTPase activated state) (*38*) (right). (**B**) Secondary structures of the most abundant tRNA^{Arg} isoacceptors, highlighting differences in primary and secondary structures compared to tRNA$^{Arg,CCG\text{-}1}$. Conserved nucleotides are greyed out to highlight nucleotide differences. Shared features involved in CNOT3 binding (marked in green in the tRNA$^{Arg,CCG\text{-}1}$ structure) are indicated by red arrows.

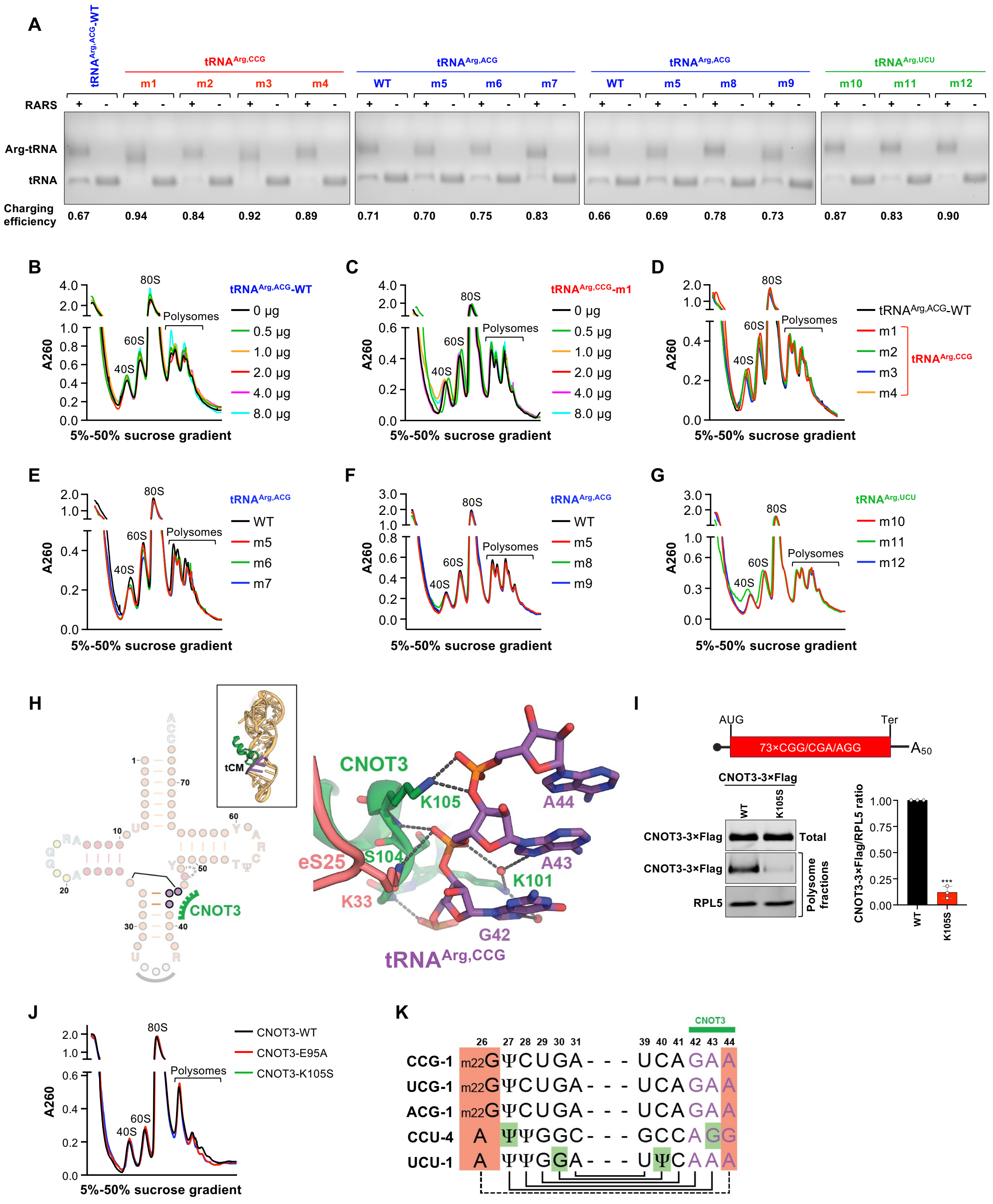

Fig. S8. Impact of P-site tRNA on CNOT3 recruitment, related to Fig. 4. (**A**) *In vitro* aminoacylation efficiency of all tRNAs used in Fig. 4. Representative data from n=2 biological replicates shown. (**B** to **G**) Sucrose density gradient profiles of *in vitro* translation reactions in Fig. 4, C through G. (**H**) Secondary structure and cartoon depictions of the interaction between

the CNOT3 tCM motif and the anticodon stem loop, highlighting the nucleotides (purple) involved in CNOT3 binding (left). Molecular details of the interaction between the tCM (green) and the ASL (purple) (right). (**I**) *In vitro* translation of the 73×CGG/CGA/AGG mRNA in lysates from cells expressing Flag-tagged wild-type CNOT3 or CNOT3 K105S, followed by western blot analysis of combined polysome fractions. n=3 biological replicates (mean ± SD shown). *P* values were calculated by student's t test. ****P*<0.001. (**J**) Sucrose density gradient profiles of *in vitro* translation reactions in (I) and Fig. 4K. (**K**) Sequences and nucleotide modifications in the anticodon stems of arginine tRNAs, highlighting the presence of G-Ψ wobble base pairs in the the ASLs of tRNAs with reduced CNOT3 recruitment.

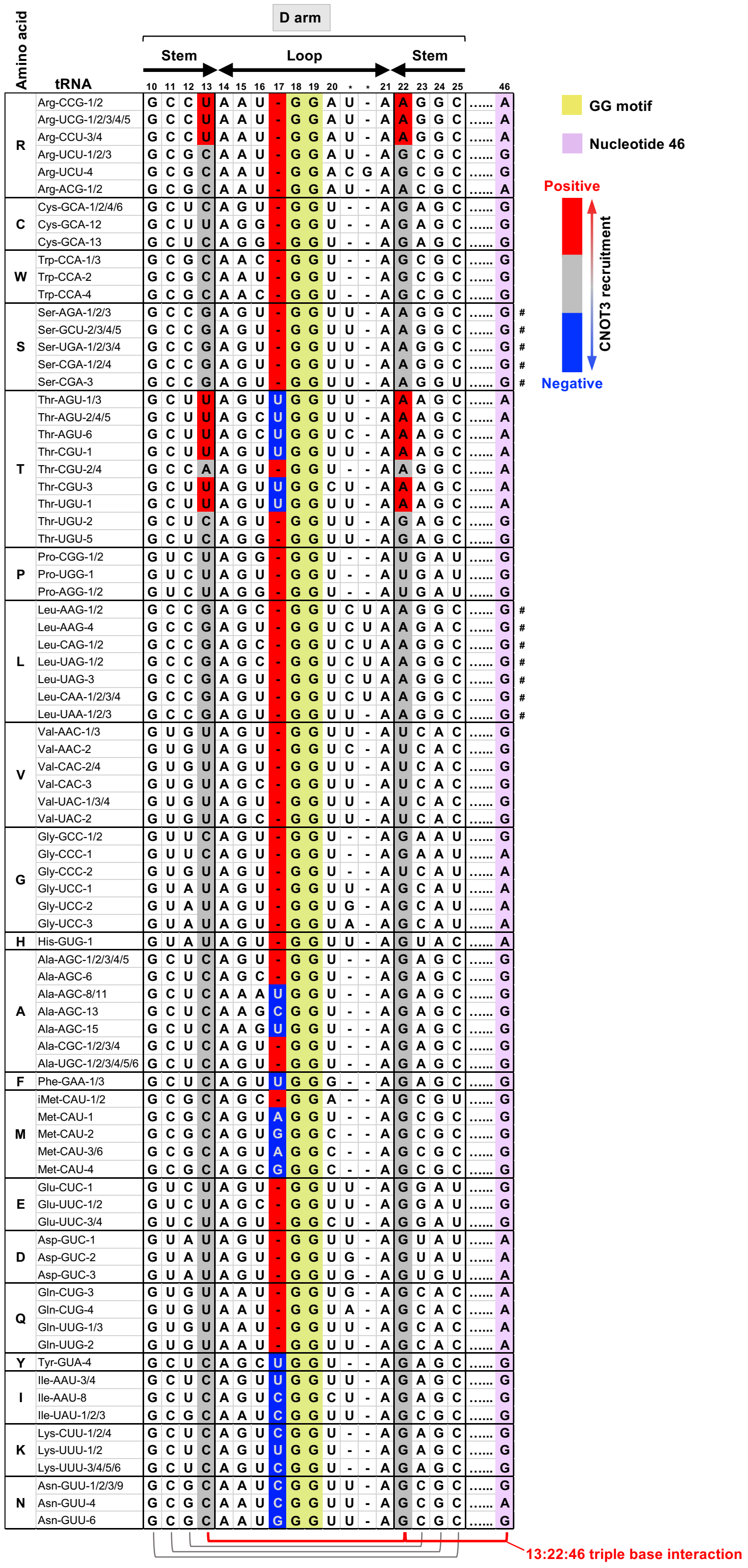

Fig. S9. D-loop sequences of human tRNAs, related to Fig. 5. Alignment of human tRNA D-arms, grouped by amino acids and arranged from highest to lowest average enrichment of each

isoacceptor group in the P-site of CNOT3-bound ribosomes. tRNAs expressed at low levels [<500 normalized counts in HEK293T tRNA sequencing data (*36*)] were excluded. The G at position 46 in Leu and Ser tRNAs does not form the triple interaction with nucleotides 13:22 because of the large variable region in these tRNAs (indicated with #).

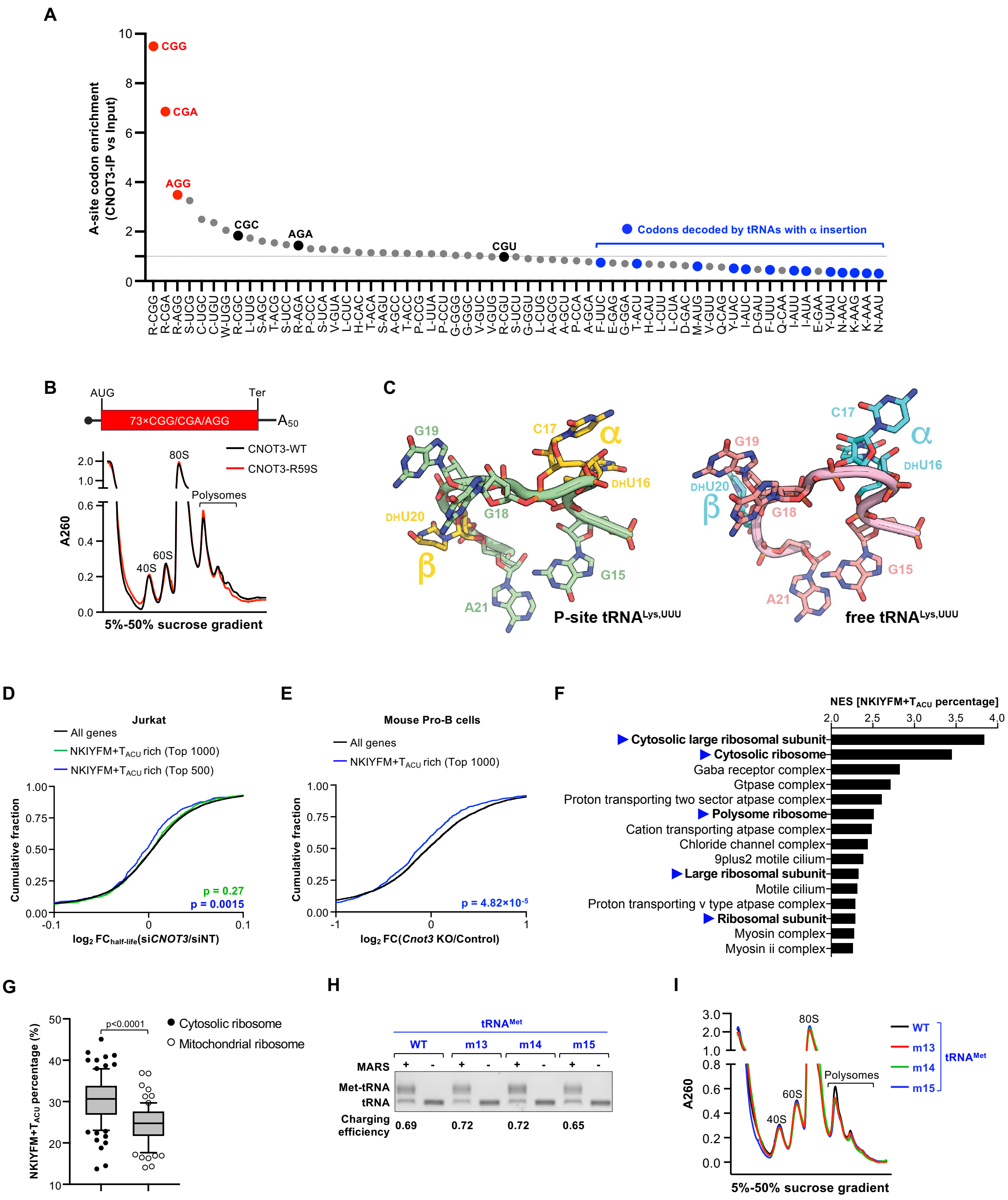

Fig. S10. Analysis of codon depletion in CNOT-bound ribosomes, related to Fig. 5. (**A**) P-site codon enrichment in CNOT3-bound ribosomes. Red and black, arginine codons; blue, codons decoded by tRNAs with α element insertions. (**B**) Sucrose density gradient profiles of *in vitro* translation reactions in Fig 5C. **(C)** Molecular models of rabbit $tRNA^{Lys,UUU}$ in the ribosomal P-site from PDB 6SGC (*48*) (left) and of the crystal structure of isolated bovine

$tRNA^{Lys,UUU}$ from PDB 1FIR (*49*) (right). (**D** and **E**) CDF plots showing the fold-change in half-lives (D) or steady-state abundance (E) of mRNAs rich in codons decoded by tRNAs with an α element insertion (N, K, I, Y, M, F, and T_{ACU}) in CNOT3-depleted Jurkat cells (*33*) or mouse pro-B cells (*34*), respectively. *P* values calculated by one-sided Wilcoxon rank sum test. (**F**) GSEA showing the top 15 genesets based on N, K, I, Y, M, F, and T_{ACU} content (all with FDR<0.25). Blue triangles indicate genesets containing cytosolic ribosomal proteins. (**G**) N, K, I, Y, M, F, and T_{ACU} content of cytosolic and mitochondrial ribosomal proteins. *P* value calculated by student's t test. (**H**) *In vitro* aminoacylation efficiency of all tRNAs used in Fig. 5L. Representative data from n=2 biological replicates shown. (**I**) Sucrose density gradient profiles of *in vitro* translation reactions in Fig 5L.

GG motif

Nucleotide 46

		D arm																		
		Stem				Loop									Stem					
	Arg tRNAs	10	11	12	13	14	15	16	17	18	19	20	*	*	21	22	23	24	25	46
Homo sapiens	**Arg-CCG**	G	C	C	U	A	A	U	-	G	G	A	U	-	A	A	G	G	C	A
	**Arg-UCG**	G	C	C	U	A	A	U	-	G	G	A	U	-	A	A	G	G	C	A
	**Arg-CCU**	G	C	C	U	A	A	U	-	G	G	A	U	-	A	A	G	G	C	A
	Arg-UCU	G	C	G	C	A	A	U	-	G	G	A	U	-	A	G	C	G	C	G
	Arg-ACG	G	C	G	C	A	A	U	-	G	G	A	U	-	A	A	C	G	C	A
Mus musculus	**Arg-CCG**	G	C	C	U	A	A	U	-	G	G	A	U	-	A	A	G	G	C	A
	**Arg-UCG**	G	C	C	U	A	A	U	-	G	G	A	U	-	A	A	G	G	C	A
	**Arg-CCU**	G	C	C	U	A	A	U	-	G	G	A	U	-	A	A	G	G	C	A
	Arg-UCU-1/2/3	G	C	G	C	A	A	U	-	G	G	A	U	-	A	G	C	G	C	G
	Arg-UCU-4	G	C	G	C	A	A	U	-	G	G	A	C	G	A	G	C	G	C	G
	Arg-ACG	G	C	G	C	A	A	U	-	G	G	A	U	-	A	A	C	G	C	A
Gallus gallus	**Arg-CCG**	G	C	C	U	A	A	U	-	G	G	A	U	-	A	A	G	G	C	A
	**Arg-UCG**	G	C	C	U	A	A	U	-	G	G	A	U	-	A	A	G	G	C	A
	**Arg-CCU**	G	C	C	U	A	A	U	-	G	G	A	U	-	A	A	G	G	C	A
	Arg-UCU-1/3	G	C	G	C	A	A	U	-	G	G	A	U	-	A	G	C	G	C	G
	Arg-UCU-2	G	C	G	C	A	A	U	-	G	G	A	C	G	A	G	C	G	C	G
	Arg-UCU-4	G	C	C	U	A	A	U	-	G	G	A	U	-	A	A	G	G	C	A
	Arg-ACG	G	C	G	C	A	A	U	-	G	G	A	U	-	A	A	C	G	C	A
Anolis carolinensis	**Arg-CCG**	G	C	C	U	A	A	U	-	G	G	A	U	-	A	A	G	G	C	A
	**Arg-UCG**	G	C	C	U	A	A	U	-	G	G	A	U	-	A	A	G	G	C	A
	**Arg-CCU-1/2**	G	C	C	U	A	A	U	-	G	G	A	U	-	A	A	G	G	C	A
	**Arg-CCU-3**	G	C	C	U	A	A	U	-	G	G	A	C	-	A	A	G	G	C	A
	Arg-UCU-1/2	G	C	G	C	A	A	U	-	G	G	A	U	-	A	G	C	G	C	G
	Arg-UCU-3	G	C	G	C	A	A	U	-	G	G	A	C	G	A	G	C	G	C	G
	Arg-ACG	G	C	G	C	A	A	U	-	G	G	A	U	-	A	A	C	G	C	A
Danio rerio	**Arg-CCG**	G	C	C	U	A	A	U	-	G	G	A	U	-	A	A	G	G	C	A
	**Arg-UCG**	G	C	C	U	A	A	U	-	G	G	A	U	-	A	A	G	G	C	A
	**Arg-CCU**	G	C	C	U	A	A	U	-	G	G	A	U	-	A	A	G	G	C	A
	Arg-UCU	G	C	G	C	A	A	U	-	G	G	A	U	-	A	G	C	G	C	G
	Arg-ACG-1/2/3/4/5/7/8	G	C	G	C	A	A	U	-	G	G	A	U	-	A	A	C	G	C	A
	Arg-ACG-6	G	C	G	C	A	A	U	-	G	G	A	C	-	A	A	C	G	C	A
Drosophila melanogaster	**Arg-UCG-1,2,4**	G	C	C	U	A	A	U	-	G	G	A	U	-	A	A	G	G	C	A
	**Arg-UCG-3**	G	C	G	C	A	A	U	-	G	G	A	U	-	A	A	C	G	C	A
	**Arg-CCU**	G	C	C	U	A	A	U	-	G	G	A	U	-	A	A	G	G	C	A
	Arg-UCU-1	G	C	G	C	A	G	A	-	G	G	A	U	-	A	G	C	G	C	G
	Arg-UCU-2	G	C	G	C	A	U	U	-	G	G	A	U	-	A	G	C	G	C	G
	Arg-UCU-3	G	C	C	U	A	A	U	-	G	G	A	U	-	A	A	G	G	C	A
	Arg-ACG	G	C	G	C	A	A	U	-	G	G	A	U	-	A	A	C	G	C	A

13:22:46 triple base interaction

Fig. S11. Alignment of metazoan arginine tRNA D-arms, related to Fig. 6.

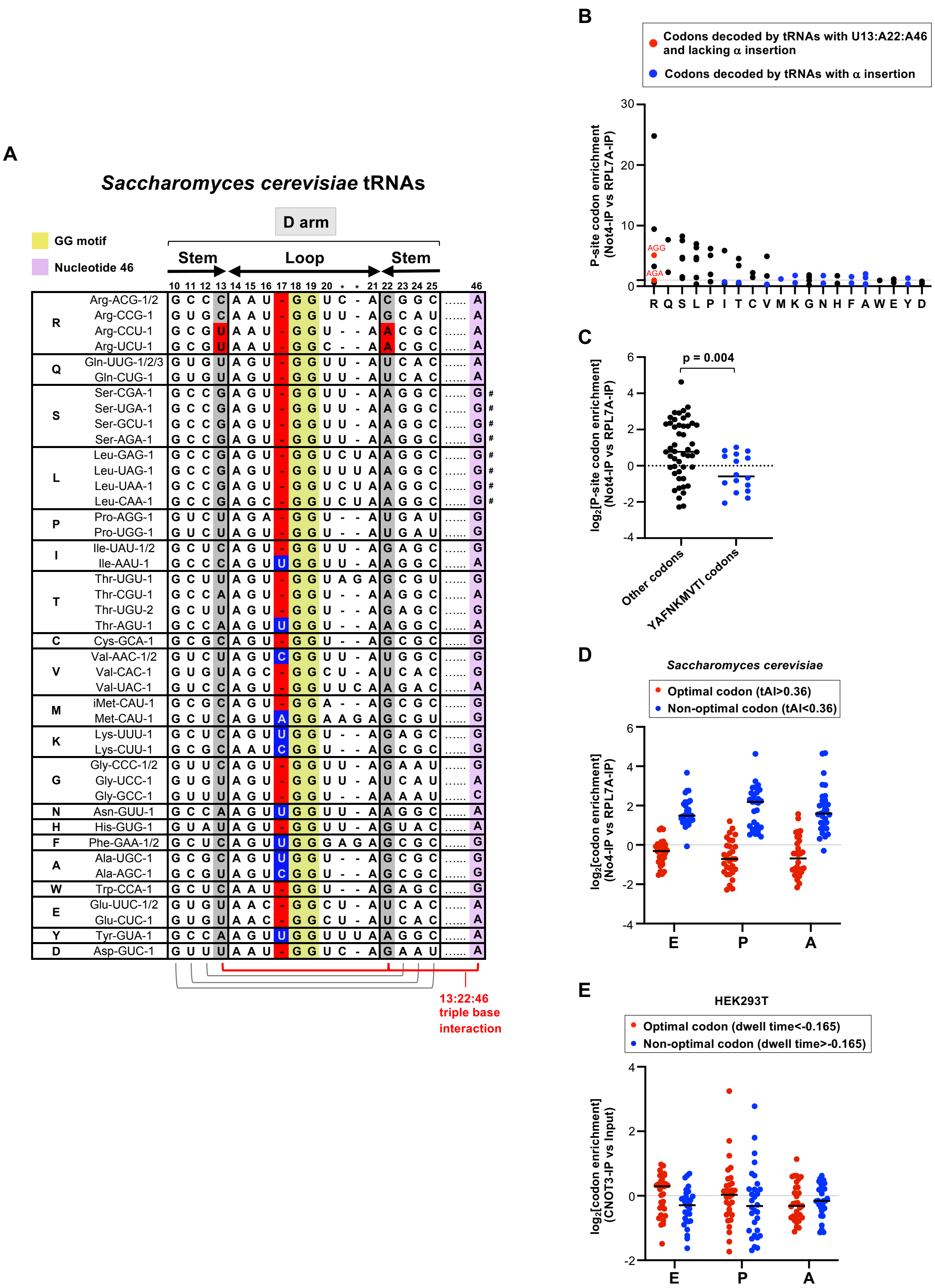

Fig. S12. Co-translational recruitment of Not5 in yeast is less dependent upon P-site tRNA identity, related to Fig. 6. (**A**) Alignment of *S. cerevisiae* tRNA D-arms. The G at position 46 in

Leu and Ser tRNAs does not form the triple interaction with nucleotides 13:22 because of the large variable region in these tRNAs (indicated with #). (**B**) Codon enrichment in the ribosomal P-site of Not4-bound ribosomes in *S. cerevisiae* (*14*). (**C**) Enrichment of codons decoded by tRNAs with or without an α element insertion in the P-site of Not4-bound ribosomes. *P* value calculated by student's t test. (**D**) Non-optimal codons are enriched in the ribosomal E-, P-, and A-sites of Not4-bound ribosomes in *S. cerevisiae*. (**E**) Non-optimal codons (defined by A-site dwell time > -0.165) are not enriched in the ribosomal E-, P-, or A-site of CNOT3-bound ribosomes in HEK293T cells.

Supplemental Table 1. Cryo-EM data collection, refinement, and validation statistics

	80S-CNOT3-tRNA$^{Arg,CGG-1}$<br>PDB 9C3H<br>EMD-45170	CNOT3-tRNA$^{Leu, TAA-1}$<br>PDB 9C3I<br>Ref. Map EMD-16052
**Data collection/processing**		
Microscope	Titan Krios	
Voltage	300 kEV	
Camera	Falcon 4	
Magnification	135,000x	
Pixel size (Å)	0.936	
Total electron exposure $e^-/Å^2$	19.15	
Exposure rate (e^-/pixel/sec)	8.39	
Number of frames	496	
Defocus range (μm)	-0.6 – (-1.9)	
Automation software	SerialEM	
Slit width (μm)	10	
Initial particle images (no.)	11907	
Final particle images (no.)	11154	
Total extracted particles	810K	
Refined particles	378K	
Final refined particles	276K	
Symmetry imposed	C2	
Map resolution (Å)		
FSC threshold 0.5	2.4 (masked), 2.8 (unmasked)	
FSC threshold 0.143	2.0 (masked), 2.3 (unmasked)	
Map resolution range (Å)	1.88 – 10	
Map sharpening B factor ($Å^2$)	74	
Map sharpening method	RELION 4	
**Model composition**		
Non-hydrogen atoms	222228	6186
Protein residues	8913	231
RNA nucleotides	2223	72
Ligands	19	0
Mg	326	0
K	94	0
Zn	8	0
Waters	9485	0
**Model Refinement**		
Refinement package	PHENIX	PHENIX
Map-Model scores		
Cross-correlation	0.81	n/a
Cross-correlation ligands	0.71	n/a
Model resolution		n/a
FSC threshold 0.5	2.31 (unmasked)	n/a
B factors ($Å^2$)		
Protein	15.45	53.30

Nucleotides	25.41	32.99
Ligand	17.41	n/a
Water	15.13	n/a
R.m.s. deviations		
Bond lengths (Å)	0.005	0.002
Bond angles (°)	0.750	0.411
Validation		
MolProbity score	1.72	1.15
CaBLAM outliers (%)	1	2.2
Clashscore	5.64	3.55
Poor rotamers (%)	1.56	0.47
C-beta deviations	0	0
EMRinger score	5.58	n/a
Ramachandran plot		
Favored (%)	97.88	98.69
Allowed (%)	2.1	1.31
Disallowed (%)	0.02	0
